# Adaptive response by an electrolyte: resilience to electron losses in a dye-sensitized porous photoanode†

**DOI:** 10.1039/d1sc00384d

**Published:** 2021-03-25

**Authors:** Frances A. Houle

**Affiliations:** Chemical Sciences Division, Lawrence Berkeley National Laboratory 1 Cyclotron Road Berkeley CA 94720 USA fahoule@lbl.gov

## Abstract

Photovoltage and photocurrents below theoretical limits in dye-sensitized photoelectrochemical solar energy conversion systems are usually attributed to electron loss processes such as dye–electron and electrolyte–electron recombination reactions within the porous photoanode. Whether recombination is a major loss mechanism is examined here, using a multiscale reaction–diffusion computational model to evaluate system characteristics. The dye-sensitized solar cell with an I^−^/I_3_^−^ redox couple is chosen as a simple, representative model system because of the extensive information available for it. Two photoanode architectures with dye excitation frequencies spanning 1–25 s^−1^ are examined, assuming two distinct recombination mechanisms. The simulation results show that although electrolyte–electron reactions are very efficient, they do not significantly impact photoanode performance within the system as defined. This is because the solution-phase electrolyte chemistry plays a key role in mitigating electron losses through coupled reactions that produce I^−^ within the photoanode pores, thereby cycling the electrolyte species without requiring that all electrolyte reduction reactions take place at the more distantly located cathode. This is a functionally adaptive response of the chemistry that may be partly responsible for the great success of this redox couple for dye-sensitized solar cells. The simulation results provide predictions that can be tested experimentally.

## Abstract 2

Interfacial electrolyte reactions in the pores of a photoanode consume electrons. The losses are offset by compensating solution-phase reactions that generate I^−^ locally, and promote efficient dye cycling and photocurrent generation.

## Graphical abstract

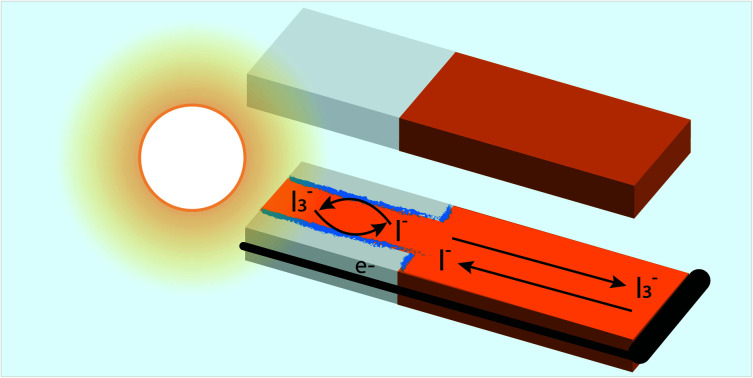

## Introduction

In photoelectrochemical solar energy conversion systems, which rely on a diffuse energy resource, any factor leading to loss of efficiency in generating electrons or chemical products must be identified and minimized. This is a significant challenge because of the complexity of the chemistry and physics involved. For example, electron losses due to recombination with chemical species in the local environment have been identified as a cause of reduction in photocurrent and photovoltage of dye-sensitized solar cells (DSCs),^1–7^ and considerable effort has been made to synthesize structures^8,9^ and chemical compositions^10–13^ that mitigate them. These elegant approaches often had less effect than hoped, signaling that the mechanisms responsible for performance losses are not fully understood. Photoelectrochemical solar energy conversion systems are highly integrated structures, which makes the design and interpretation of experimental studies that uniquely examine loss processes in them challenging. In this work we take an alternative approach, using a physically-based, detailed multiscale reaction–diffusion computational model that allows the inclusion and omission of well-defined mechanistic steps to pinpoint which of them cause losses, and which do not.

**Fig. 1:**
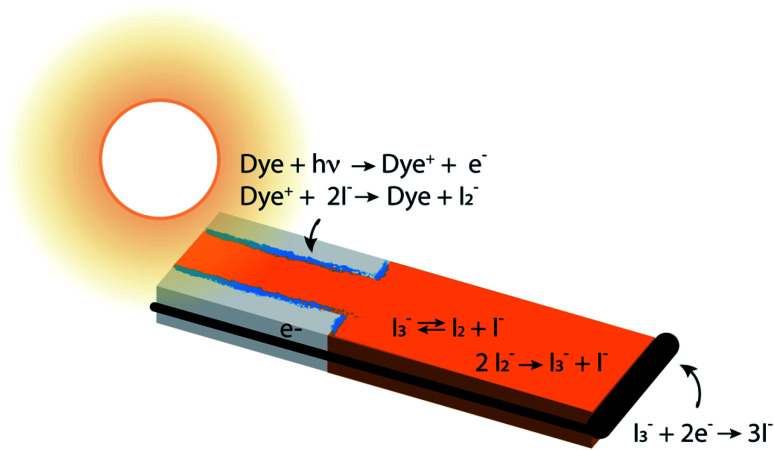
Cross section through a single pore in a DSC photoanode illuminated from the anode contact side (pore bottom) by sunlight, delineating anodic, bulk electrolyte and cathodic regions and showing the locations of the major chemical reactions. The aspect ratio is not to scale – typical pore diameters are in the range of 10 nm, and the photoanode and overall device thicknesses are usually in the range of 10–20 and 40–80 μm, respectively. The gray regions are TiO_2_ which is transparent to visible light, the blue regions are an adsorbed dye layer, the iodine-containing electrolyte is orange, and the electron conductor and cathode are black. A wire connects the bottom of the anode pore to the cathode, where electrolyte reduction reactions take place. The concentrations of the majority electrolyte ions, I^−^ and I_3_^−^, are connected *via* an equilibrium reaction. When the DSC is operating, reduction of the oxidized dye, Dye^+^, leads to a significant local depletion in I^−^ concentration within the pore. The I_2_^−^ formed as a result of I^−^ reactions with Dye^+^ undergoes a disproportionation reaction to regenerate I^−^ and I_3_^−^. This leads to a buildup of I_3_^−^ in the photoanode pores, forming a concentration gradient that drives its diffusion toward the cathode, where it is reduced to 3I^−^.

DSCs are ideal model systems for this type of study because their function is relatively simple, and they enjoy a detailed yet not fully settled literature on the mechanisms of electron losses. Typical DSCs are constructed of photoanodes that are commonly a porous network of TiO_2_ nanoparticles in contact with a conductor, paired with a counterelectrode.^14^ The nanoparticle surfaces are coated with dyes that inject electrons into the TiO_2_ after photoexcitation, and the network is infiltrated with an electrolyte containing a redox couple such as I^−^/I_3_^−^ that circulates electrons through the system. The chemical reactions taking place in the system are illustrated in Fig. 1. I_3_^−^ is formed by disproportionation of I_2_^−^ in the photoanode during operation, and diffuses through bulk electrolyte to a cathode, where it is reduced to I^−^. The I^−^ carries electrons back to the photoanode thereby completing the circuit. Transient photoresponse measurements on DSCs have been made to determine electron lifetimes following injection from the dyes, and extract information on electron loss rates in open circuit structures.^15–19^ According to these investigations, transient decay is attributed to interfacial processes such as dye–electron recombination and electrolyte–electron recombination in the photoanode.

Of the two recombination channels, dye–electron recombination (sometimes referred to as back electron transfer) has been determined to be relatively unimportant, whereas electrolyte–electron interactions are thought to be the major loss process.^1,15–17,20–23^ Two species present in the I^−^/I_3_^−^ electrolyte have been identified as potential reactants with electrons at the photoanode–dye–electrolyte interface. In early studies, it was proposed that I_3_^−^ is reduced by trapped electrons, similar to the reaction that occurs at the system's cathode.^7,15^ In fact, further investigations have shown that I_2_, formed in the bulk electrolyte, is the most likely solution-phase species to scavenge photogenerated electrons, generating I_2_^−^ as a product.^24,25^ However, because the equilibrium constant *K*_eq_ for the reaction I_3_^−^ ⇄ I_2_ + I^−^ strongly favors I_3_^−^, the I_2_ concentration has been expected to be low in the presence of I^−^. Accordingly, it is thought that the recombination rate with I_2_ would be slow, and electrolyte–electron recombination reactions would be likely to be a minor influence on the overall system function.

This suggestion is reasonable, but it carries an underlying assumption that equilibrium among the iodine and iodide species is maintained and overwhelmingly forms I_3_^−^ under all conditions.^26^ The chemistry of this redox couple is complex,^12^ however, and both interfacial and solution phase reactions contribute to the instantaneous local concentrations of iodine-containing species in the electrolyte (Fig. 1). In particular, enrichment of I_3_^−^ and depletion of I^−^ in the pores of a working photoanode has been recognized in previous studies.^27,28^ This could affect the local concentration of I_2_ (an electron scavenger) *via* shifting the I^−^/I_3_^−^ ratio away from its equilibrium value. Such a shift could provide significant quantities of I_2_ in the pores, potentially increasing the importance of electron recombination beyond what has been estimated. Establishing experimentally whether or not this occurs is very challenging because of the inaccessibility of nanometer-scale information on the spatially-dependent chemical composition and electron density distributions in nanoporous DSC photoanodes during operation.

Detailed, physically-based multiscale reaction–diffusion calculations offer a means to overcome this limitation and understand in a holistic way how electrolyte chemistry, photoexcitation in the anode, and electron losses are coupled. In the present work, simulations are used to address a simple question: *are electron*–*electrolyte recombination reactions in the photoanode uniquely responsible for performance limitations of the full DSC system*? The results show that, within the model structure used, the answer is *no*, and that an unexpected mechanism is involved. The calculations provide evidence that homogeneous electrolyte chemistry plays a key role in controlling the system's response to light *operando* and may be part of the reason that the I^−^/I_3_^−^ redox couple has been so successful as a component of DSC systems. Specifically, the solution-phase reactions confer a notable resilience on the system by providing redundancy in the generation of I^−^, a reagent crucial to Dye^+^ reduction and therefore the efficiency of dye regeneration. This is a local, adaptive response of the electrolyte, driven by changing conditions in the photoanode and by the participation of electrolyte–electron recombination reactions. The idea that chemical reactions are central to adaptive behaviors of systems is not new, however reported studies generally examine the adaptivity of molecular structure to external stimuli. In the present work, the chemistry enables *adaptive function* that mitigates what otherwise would be pure losses in the photoanode.

## Model construction

The multiscale, reaction–diffusion framework used in this work has been constructed using experimental data available in the literature, permitting a connection to be drawn between events occurring at the nanometer scale and full system function at the 10 s of microns scale *via* the system's master equation.^29^ The framework's basic characteristics, which have been published previously,^28^ are summarized. How the starting framework has been extended to include electron recombination reactions and actively maintained equilibria as local concentrations change is described in detail.

All simulations were performed using the kinetics simulation package Kinetiscope,^30^ which is a type of kinetic Monte Carlo calculation that uses particles to represent molecular species, and generates a solution to the master equation for the system using stochastic techniques.^31,32^ This package builds on algorithms originally described for single compartments^33,34^ to support full 3-D reaction–diffusion kinetics with local spatial resolution. The simulations span nm–micron as appropriate, and generate a complete time history for spatially-resolved concentrations of all species in the system. The particle-based simulation technique permits use of non-chemical species, denoted as marker species, to record the occurrence of specific types of reactions in specific locations. Recent examples of how this computational technique aids in analysis of the simulation results are presented elsewhere,^28,35,36^ details pertinent to how it is implemented in this work are presented in several sections of the ESI.†

### Base model framework

**Fig. 2:**
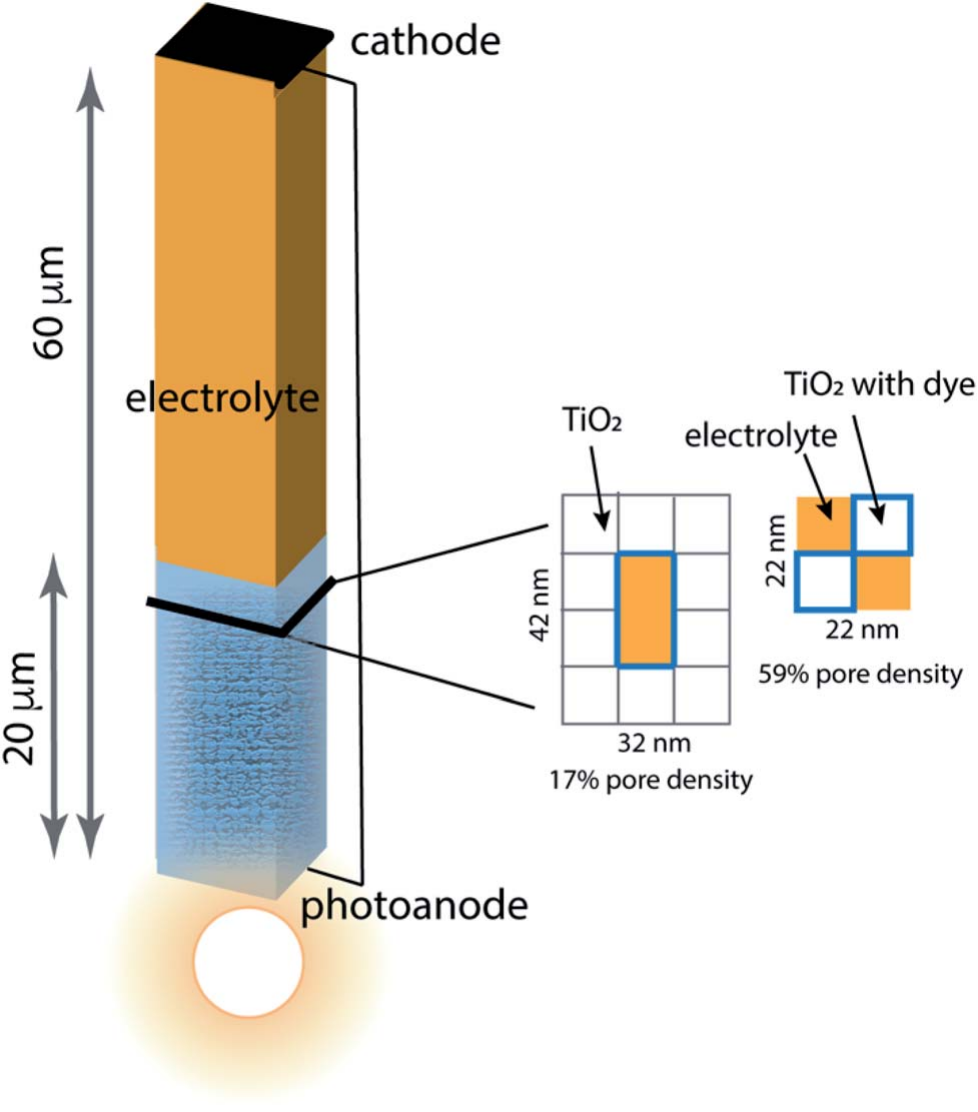
Schematic of the model architecture using the color scheme from Fig. 1. The photoanode is represented using a single pore surrounded by stacks of 10 nm × 10 nm × 10 nm TiO_2_ nanoparticles that are coated with dye, filled with electrolyte and in contact with bulk electrolyte. Two limiting pore architectures are considered, and their cross-sectional structure is shown.

Porous photoanodes in DSCs are constructed of TiO_2_ nanoparticles arranged in a complex, randomly packed structure. In previous work,^28^ two limiting model architectures were defined to represent this structure in a simplified way that permits connection between function and geometry to be made. Briefly, the system is represented by a single pore of well-defined length and cross section, as illustrated in Fig. 2. The interior walls of the pore are covered with a generic photosensitizing dye that has typical light absorption and excitation properties. Because a nanoporous photoanode will have a broad range of pore sizes and proximities due to the random nature of nanoparticle packing, two limiting pore density cases are considered: 17% pore density, and 59% pore density. In both cases the pores are 20 μm long, filled with an I^−^/I_3_^−^ electrolyte, and separated from the cathode by a 40 μm thick layer of bulk electrolyte. The mnemonic used to name them is P̲ore D̲ensity *x*%–pore length, for example the 59% pore density–20 μm pore length architecture is called PD59-20. The anode contact at the bottom of the pore is connected by a zero-resistance wire to the cathode contact above the electrolyte layer. The TiO_2_ anode material is modeled as 10 nm particles in linear stacks surrounding the pores. The 17% density case has a 10 × 20 nm cross sectional area fully surrounded by TiO_2_, and the 59% density case is a 10 × 10 nm cross sectional checkerboard with wraparound diffusion paths to make a boundaryless system. Neglect of tortuosity is not likely to significantly affect the calculations since its main influence would be to slow down overall diffusion rates within the anode. The dye sensitizer molecules are assumed to be in a uniform coating 1 nm thick on all surfaces in contact with the electrolyte, with a concentration of 4.4 × 10^20^ mol cm^−^^3^.^14^ The electrolyte composition has an initial value of 3.01 × 10^19^ mol cm^−3^ I_3_^−^ and 3.01 × 10^20^ mol cm^−3^ I^−^ throughout the system, and diffusion and reaction coefficients for an acetonitrile solution. Electrons injected into the TiO_2_ after photon absorption by the dye diffuse to an anode contact, and are conducted to the cathode where reduction of I_3_^−^ to form I^−^ takes place. Diffusion of the I^−^ back to the anode pore serves to complete the circuit. The reaction steps and rate constants used in this work build on the base model, and are presented in Table 1. The base model is processes 2–4 and 7–10. Processes 1, 5, 6, and 11 are new in this work.

**Table 1:** List of reaction steps and kinetic parameters used in the simulations

Process	Step	Rate constant
1. Equilibrium	I_3_^−^ → I_2_ + I^−^	3 × 10^3^ s^−1^^a^
	I_2_ + I^−^ → I_3_^−^	5 × 10^−12^ cm^3^ mol^−1^-s^−1^^b^
2. Anode–electrolyte interface reaction	Dye + *hν* → Dye^+^ + electron	1–25 s^−1^ (ref. 14)^c^
	Dye^+^ + I^−^ → Dye–I	1 × 10^−10^ cm^3^ mol^−1^-s^−1^^d^
	Dye–I + I^−^ → Dye + I_2_^−^	1 × 10^−10^ cm^3^ mol^−1^-s^−1^^d^
3. Electrolyte reaction	2I_2_^−^ → I^−^ + I_3_^−^	3.8 × 10^−11^ cm^3^ mol^−1^-s^−1^ (ref. 12)
4. Cathode–electrolyte interface reaction	I_3_^−^ + 2 electrons → 3I^−^	1 × 10^−20^ cm^6^ mol^−2^-s^−1^^d^
5. Interfacial electron recombination	I_2_ + e^−^ → I_2_^−^	6.64 × 10^−18^ cm^3^ mol^−1^-s^−1^ (ref. 25)
6. Interfacial I_2_ reaction	I_2_ + I^−^ → I_3_^−^	5 × 10^−12^ cm^3^ mol^−1^-s^−1^^b^
7. Electron diffusion	Electrolyte interface to anode TiO_2_ matrix	2 × 10^−4^ cm^2^ s^−1^ (ref. 14)^e^
	In TiO_2_ matrix toward anode contact	2 × 10^−4^ cm^2^ s^−1^ (ref. 14)^e^
	From anode contact to cathode contact	1 cm^2^ s^−1^^a^
	From cathode to electrolyte interface	2 × 10^−4^ cm^2^ s^−1^ (ref. 14)^e^
8. Electrolyte ion diffusion	I_3_^−^ in electrolyte	1 × 10^−5^ cm^2^ s^−1^ (ref. 37 and 38)^e^
	I_2_^−^ in electrolyte	1 × 10^−5^ cm^2^ s^−1^ (ref. 37 and 38)^e^
	I^−^ in electrolyte	2 × 10^−5^ cm^2^ s^−1^ (ref. 37 and 38)^e^
9. Ion adsorption at interface	I^−^ to anode–electrolyte interface	2 × 10^−5^ cm^2^ s^−1^ (ref. 37 and 38)^e^
10. Ion desorption from interface	I_3_^−^ from anode–electrolyte interface	1 × 10^−5^ cm^2^ s^−1^ (ref. 37 and 38)^e^
	I_2_^−^ from anode–electrolyte interface	1 × 10^−5^ cm^2^ s^−1^ (ref. 37 and 38)^e^
11. Electrolyte I_2_ diffusion	I_2_ in electrolyte	1 × 10^−5^ cm^2^ s^−1^ (ref. 37 and 38)^e^

a Calculated from measured *K*_eq_ and Smoluchowski equation for the reverse step.

b Calculated from the Smoluchowski equation.

c This represents a complex sequence of excitations, energy transitions, relaxations and charge injection, all taking place on the ps–μs timescale. The overall frequency of this step is in the range of fractions of seconds, however, because of the low light intensity.

d Assumed value for rate constant, selected to be very large.

e Estimated from range in cited references.

### Modeling electron recombination with I_2_

In order to examine the influence of electron recombination with I_2_, a source for it has to be explicitly included in the electrolyte chemistry. The initial concentration of I_3_^−^ relative to I^−^ and I_2_ is governed by the equilibrium constant *K*_eq_ in acetonitrile, 1 × 10^6^ M^−1^.^25,39^ This value is at the low end of the range in the literature, 10^6^–10^7^ M^−1^,^12,25,39–41^ and consistent with the analysis that generated the I_2_ recombination rate coefficient.^25^ (The sensitivity to using this low value is not significant, as discussed in the ESI, Section 2.†) When the electrolyte is formulated, the I_2_ concentration is extremely low because it is added to an excess of I^−^ and is converted essentially entirely to I_3_^−^. If the initial I^−^ and I_3_^−^ ion concentrations are unaltered during system operation, equilibrium is maintained and the I_2_ concentration should remain low. However, during operation the I^−^/I_3_^−^ ratio decreases in the pores,^27,28^ which can increase the I_2_ concentration according to the reversible reactions, shown in Table 1, process 1. Rate constants for these forward and reverse steps in acetonitrile have not been reported. The Smoluchowski equation is used to estimate the rate constant for I^−^ + I_2_ → I_3_^−^ assuming van der Waals radii of 0.2 nm for the reactants, and a diffusion coefficient of 10^5^ cm^2^ s^−1^ (Table 1). The value of 5 × 10^−12^ cm^2^ s^−1^ is about half of the value measured in protic solution.^42^ The dissociation rate constant is calculated using it, and *K*_eq_. I_2_ is assumed to diffuse freely throughout the electrolyte, and from the electrolyte into the interfacial region where the dye is located (Table 1, process 11). Electrons are initially injected by excited dyes in this region, and are assumed to be available for recombination with electrolyte species when they are present at the interface. Electrons can diffuse into the TiO_2_ bulk with a rate determined by the electron concentration gradient between the interface and the bulk and their diffusion coefficient. Once in the bulk, they are no longer available for recombination with I_2_. The energetic state of the electrons (conduction band *vs.* mid-gap states) is important,^4,43^ but not considered in this model because insufficient information is available about the relative concentrations of these states and their reactivity toward I_2_ in the interfacial region. The appropriate kinetics can be added readily as experimental or theoretical data are published.

The details of how the electron recombination with I_2_ should be described are not fully known,^4^ so three scenarios are examined in this study. Scenario 1 (Sc1) assumes that electron transfer to I_2_ to form I_2_^−^ is an elementary step, with the measured rate constant of 6.64 × 10^−18^ cm^3^ mol^−1^-s^−1^,^25^ which is significantly slower than the values of 10^−15^–10^−16^ cm^3^ mol^−1^-s^−1^ estimated from full system models.^27^ This scenario is represented as process 5 in Table 1. Scenario 2 (Sc2) assumes that process 5 competes with reaction between I^−^ and I_2_ at the interface (process 6), which could potentially reduce the extent of electron recombination. The rate coefficient is taken to be the same value as in solution (process 1). As a control, scenario 3 (Sc3) assumes no I_2_ formation, which corresponds to the previously published base case^28^ described above.

### Performance scaling with increasing excitation

The base case from earlier work used a dye excitation frequency of 1 s^−1^ (process 2, Table 1), which is typical for 1 sun.^15,44^ Higher levels of excitation leading to higher rates of electron generation and recombination have been found to impact photoanode function.^11,27,45^ Accordingly, the 1 sun case is compared to 5×, 10× and 25× higher excitation levels for all 3 scenarios. These are represented in the model using the dye excitation step in process 2, Table 1, with rate constants of 5 s^−1^, 10 s^−1^ and 25 s^−1^. Physically, this would correspond to using more efficient dyes or use of concentrated light, or both.

## Results

Simulations were performed for both DSC architectures (Fig. 2) with 4 excitation frequencies and 3 recombination scenarios, a total of 24 data sets. Each simulation was allowed to run to 0.8 s, corresponding to steady state for nearly all runs. This time is relatively short, and is determined by the establishment of steady state gradients in the system which has very thin (1 micron) electrodes. Charging up thicker, more realistic electrode substrates is too expensive computationally, but would yield a more realistic time to reach steady state and potentially result in lower average electron densities in the system.

The simulation outputs provide time histories for I_3_^−^, I^−^, I_2_, electrons, and neutral and oxidized dyes for all systems. In addition, spatiotemporal maps for marker species provide insights to interactions and events that underly the composition *vs.* time results. Referring to Table 1, markers track each occurrence of I_2_ formation (process 1) and electron recombination by I_2_ (process 5) in Sc1 and Sc2, interfacial I_3_^−^ formation (process 6), in Sc2, and disproportionation (process 3), excitation and Dye^+^ reduction (process 2), and I_3_^−^ reduction at the cathode (process 4), in all 3 scenarios. The full set of simulation results including the markers is analyzed to understand how each scenario affects system performance.

### Effect of architecture, excitation and recombination on electrolyte composition

**Fig. 3:**
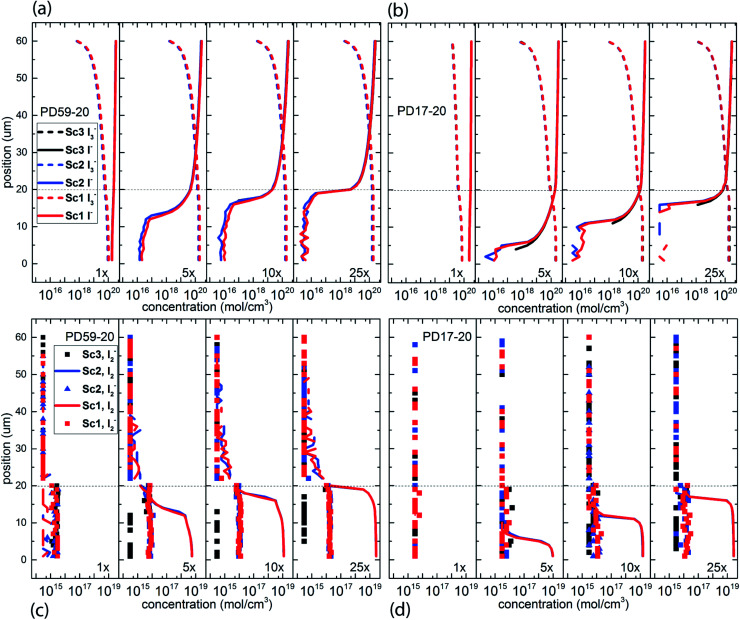
Electrolyte composition at 0.8 s (close to steady state) as a function of position within the system. The photoanode contact is at 0 μm, the interface between the photoanode and bulk electrolyte is at 20 μm, and the cathode is at 60 μm. (a) I^−^ and I_3_^−^ profiles for PD59-20, (b) I^−^ and I_3_^−^ profiles for PD17-20, (c) I_2_^−^ and I_2_ profiles for PD59-20, (d) I_2_^−^ and I_2_ profiles for PD17-20.

The electrolyte composition through the full thickness of both architectures at 0.8 s is shown in Fig. 3, and full maps are shown in the ESI, Fig. S2–S9.† The I^−^ and I_3_^−^ profiles in Fig. 3a and b are nearly the same for all 3 scenarios for each architecture, showing accumulation of I_3_^−^ and deep depletion of I^−^ in the anode pores, and depletion of I_3_^−^ near the cathode. The concentration polarization is modest at 1 s^−1^ excitation frequency, and increases significantly with increasing excitation frequency. I^−^ is more deeply depleted in PD59-20 because of the larger surface/volume ratio in the pores.^28^Fig. 3c and d show the corresponding I_2_ and I_2_^−^ populations for all 3 scenarios. It is evident that as the I^−^ concentration is reduced, I_3_^−^ is driven toward I_2_ and I^−^ formation. I_2_^−^ is formed by either I_2_ recombination with electrons from TiO_2_ (scenarios 1 and 2) or by reaction of I^−^ with oxidized dyes at the anode–electrolyte interface (all scenarios). I_2_^−^ undergoes a second order disproportionation reaction (Table 1, process 3) to regenerate I_3_^−^ and I^−^ within the pores. As shown in ESI, Fig. S10,† the total number of disproportionation events is significantly increased when electron recombination occurs, constituting a local source of I^−^ in addition to the cathodic reactions. The I_3_^−^ formed in process 3 can dissociate to form still more I_2_ and I^−^ within the pore.

In Sc3, where electron recombination does not occur, I_2_^−^ is formed only in regions where I^−^ is present to react with oxidized dyes on the pore walls. Since I^−^ is depleted in the pore and transport from the cathode is not fast enough to replace it, I_2_^−^ formation and disproportionation become progressively confined to the top of the photoanode (ESI, Fig. S10†). Under Sc1 and Sc2, on the other hand, disproportionation is spread throughout the pore volume. The concentrations of both I_3_^−^ and I^−^ in the electrolyte are reduced under Sc2, relative to Sc1. In the PD59-20 architecture, 25% of the I_3_^−^ is located at the TiO_2_–electrolyte interface where it is formed by the interfacial I_2_ reaction, Table 1 process 6. In the PD17-20 architecture, 15% of the I_3_^−^ is at the interface due to the smaller surface/volume ratio. In both cases, I_3_^−^ accumulates at the interface after it is formed because its diffusion into the bulk electrolyte is gradient-driven and therefore slow.

**Fig. 4:**
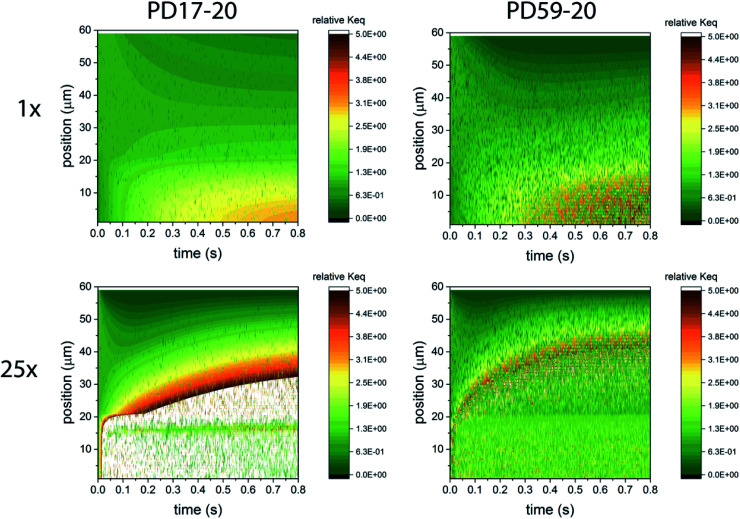
Maps of the ratio ([I_3_^−^]/[I^−^][I_2_])/*K*_eq_ as a function of position and time in the electrolyte, as a measure of deviation from expected equilibrium concentrations under Sc2. The photoanode contact is at 0 μm, the interface between the photoanode and bulk electrolyte is at 20 μm, and the cathode is at 60 μm. In the simulations 1 particle represents 3 × 10^14^ mol cm^−1^.^3^ Whenever the ion concentration is 0 particles, it is set to a value of 6 × 10^13^ mol cm^−3^ to permit these ratios to be calculated. (a) 1 s^−1^ excitation frequency, PD17-20, (b) 25 s^−1^ excitation frequency, PD17-20, (c) 1 s^−1^ excitation frequency, PD59-20, (d) 25 s^−1^ excitation frequency, PD59-20. Data for Sc1 are nearly indistinguishable from this set.

In considering the relative concentrations of I_3_^−^, I^−^ and I_2_ in an operating DSC, the question arises whether their balance is controlled only by their rapidly maintained equilibrium in solution as would be the case for a buffer. This would be expected if the kinetics allow the electrolyte to respond on a timescale that is fast relative to the interfacial reactions. To answer this question, the instantaneous concentration ratio [I_3_^−^]/[I^−^][I_2_] was normalized to *K*_eq_ as a function of position and time in the PD17-20 and PD 59–20 systems for the 1 s^−1^ and 25 s^−1^ excitation frequency cases, Sc2, as shown in Fig. 4. If equilibrium is maintained and controls all relative concentrations in the electrolyte, then the normalized value should be close to 1. If it is much less than 1, then the system is locally depleted in I_3_^−^; if it is much greater than 1, the system is locally depleted in I_2_ and/or I^−^. It is clear that the equilibrium reaction does not consistently control relative concentrations except possibly for within the pore of the PD59-20 architecture at a 25 s^−1^ excitation frequency. Rather, the electrolyte is driven away from equilibrium and it is the combination of the interfacial and solution phase reactions that controls the electrolyte composition during DSC operation.

### Photocurrent and photovoltage with and without electron recombination

**Fig. 5:**
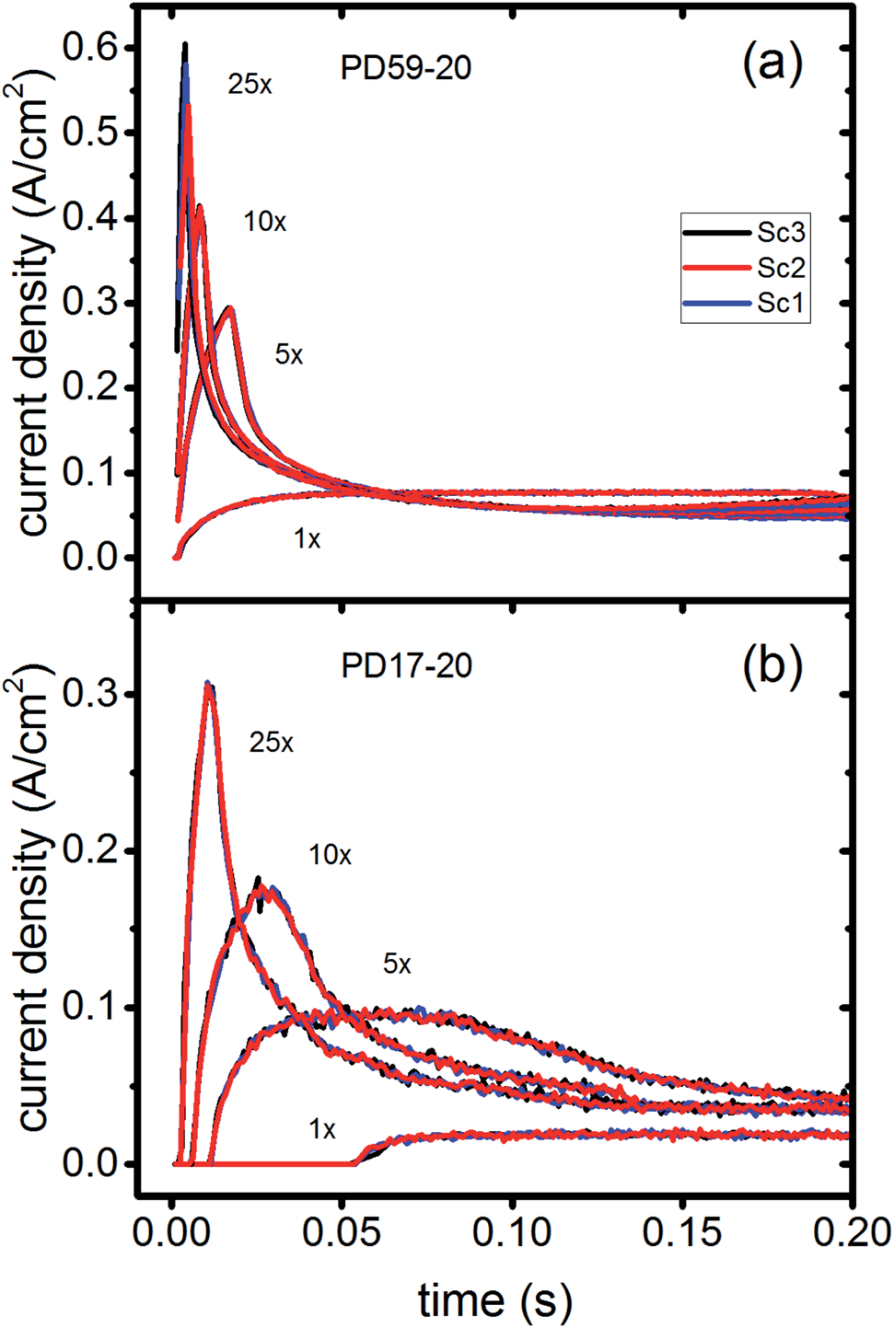
Current densities for all excitation frequencies to 0.2 s (a) PD59-20; (b) PD17-20.

In DSCs, current flow is the result of the reduction of I_3_^−^ that is formed in the photoanode. I_3_^−^ diffuses to the cathode to form 3I^−^, which then diffuse back to the photoanode. Predicted current densities are calculated by taking the derivative of the accumulating I_3_^−^ reduction event markers *vs.* time.^28^Fig. 5 presents the calculated current densities for the three scenarios and both architectures, for all excitation frequencies. For clarity only data to 0.2 s are shown, data to 0.8 s are in ESI, Fig. S11.† Photocurrent transients are predicted for all cases except PD17-20 at 1 s^−1^ excitation frequency.

**Fig. 6:**
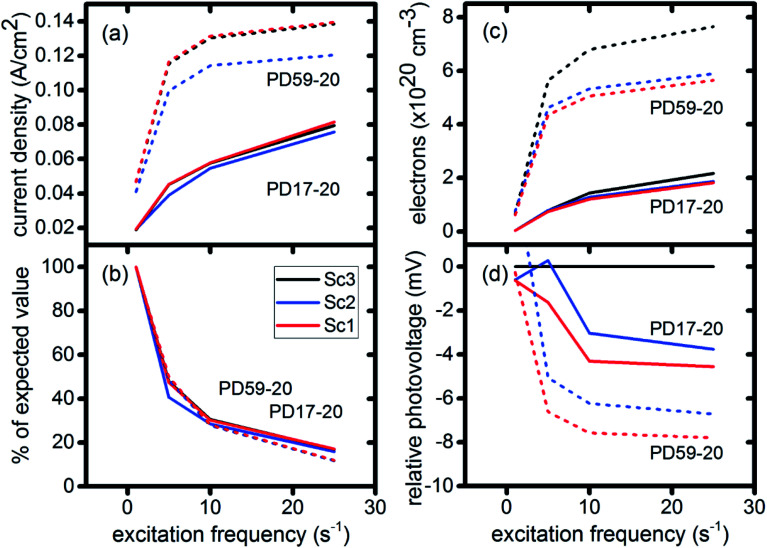
Electrical performance characteristics for both architectures and all three scenarios as a function of excitation frequency. (a) Calculated current densities at 0.8 s; (b) percent of expected current density values if they had scaled linearly with excitation frequency; (c) electron densities in the TiO_2_, averaged from top to bottom of the photoanode; (d) photovoltage relative to Sc3 (no electron recombination).

The current densities predicted for all three scenarios are similar up to 0.15 s for both architectures. The steady state/near steady state current densities for all cases calculated at 0.8 s are plotted in Fig. 6a, showing that they are similar in value for all 3 scenarios for both pore architectures. Although Sc1 and Sc3 have nearly identical current densities, that for Sc2 is reduced. This is a consequence of allowing adsorption of I_3_^−^ on the pore walls in scenario 2, which reduces [I_3_^−^] in the electrolyte. The surface to volume ratio is larger for PD59-20, so the effect is more pronounced.^28^ Both Fig. 5 and 6a show that the highly efficient electron recombination reactions and I_2_ reactions with I^−^ at the photoanode–electrolyte interface do not influence the photocurrent, and Fig. 6b shows that in all cases photocurrent scales the same way with excitation frequency. This is a surprising result: from the literature, electron recombination is expected to reduce photocurrent, and further addition of an I_2_–I^−^ recombination path that competes with electron recombination by I_2_ might have been expected to be neutral or perhaps increase current density.

Fig. 6c shows the calculated electron densities at 0.8 s in the photoanode. The electron densities are essentially the same for all 3 scenarios in the PD17-20 architecture, while electrolyte–electron recombination reduces them by about 15% in the PD59-20 architecture (Sc1 and Sc2). The electron densities can be used to calculate the relative photovoltage Δ*V* to evaluate to what extent recombination affects it. An absolute open or closed circuit photovoltage requires knowledge of the dark electron population, which the literature indicates can vary over many orders of magnitude.^18,44,46^ There is no accepted typical value to assume for the present work. The relative photovoltage, Δ*V*^ab^, can be estimated, however, from the change in electron density *D*_e_ in state b relative to that in state a. It is expressed as:1
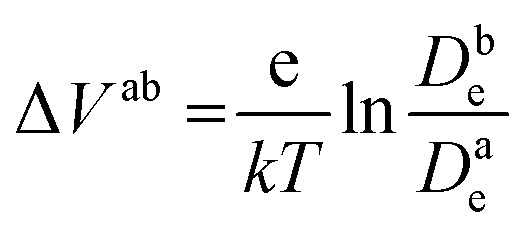
where *T* is assumed to be 298 K and here state a is Sc3 (no electrolyte–electron recombination) and state b is Sc1 or Sc2, at each excitation frequency. Fig. 6d shows that the relative photovoltage decreases between 1× and 5× excitation frequencies, but then is essentially flat. Typical measured open circuit potentials are in the range of 600 mV. For both architectures, electron recombination under both scenario 1 and scenario 2 results in a small loss in relative potential compared to the no-recombination case, scenario 3. However, the current densities do not follow this trend, indicating that the overall resistance of the system has decreased as a result of including electron recombination.

### Interfacial chemistry

**Fig. 7:**
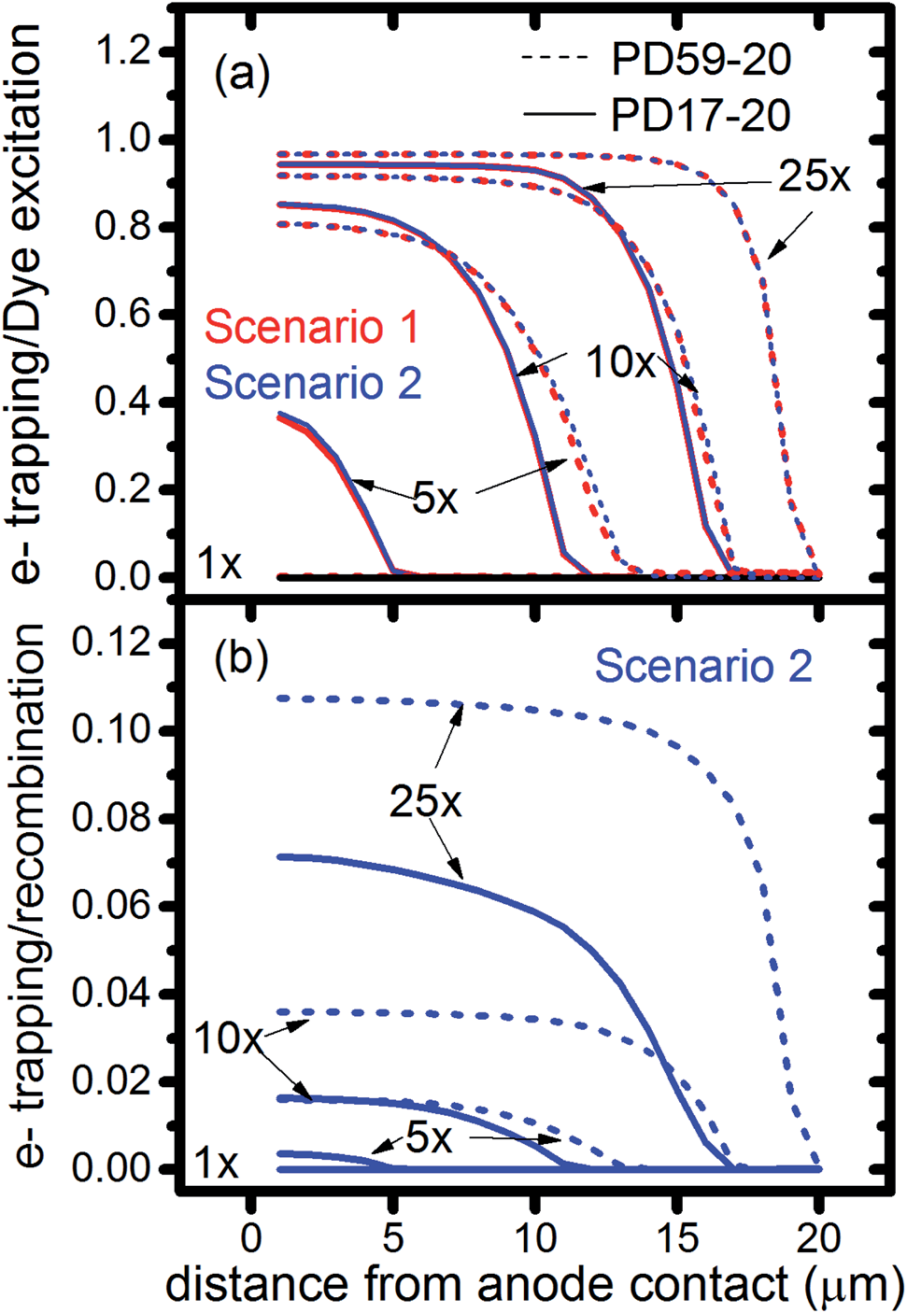
Ratios of cumulative events as a function of position in the photoanode after 0.8 s, as monitored by markers in the reaction steps. (a) Electron recombination with I_2_ relative to dye excitation for Sc1 and Sc2, (b) electron recombination with I_2_ relative to interfacial formation of I_3_^−^ from I_2_ and I^−^.

The interfacial electron recombination and I_2_ recombination reaction frequencies mirror the electrolyte composition as a function of position in the photoanode pores, shown in Fig. 3. The abundance of I_2_ present in the electrolyte provides a means for these reactions to become dominant events in the photoanodes. As shown in Fig. 7a, recombination under Sc1 and Sc2 consumes most of the electrons generated by photoexcitation at 25 s^−1^ excitation frequency, with an architecture-dependent spatial distribution. Fig. 7b shows that under Sc2, recombination of I_2_ and I^−^ to form I_3_^−^ dominates over electron recombination at all excitation frequencies.

**Fig. 8:**
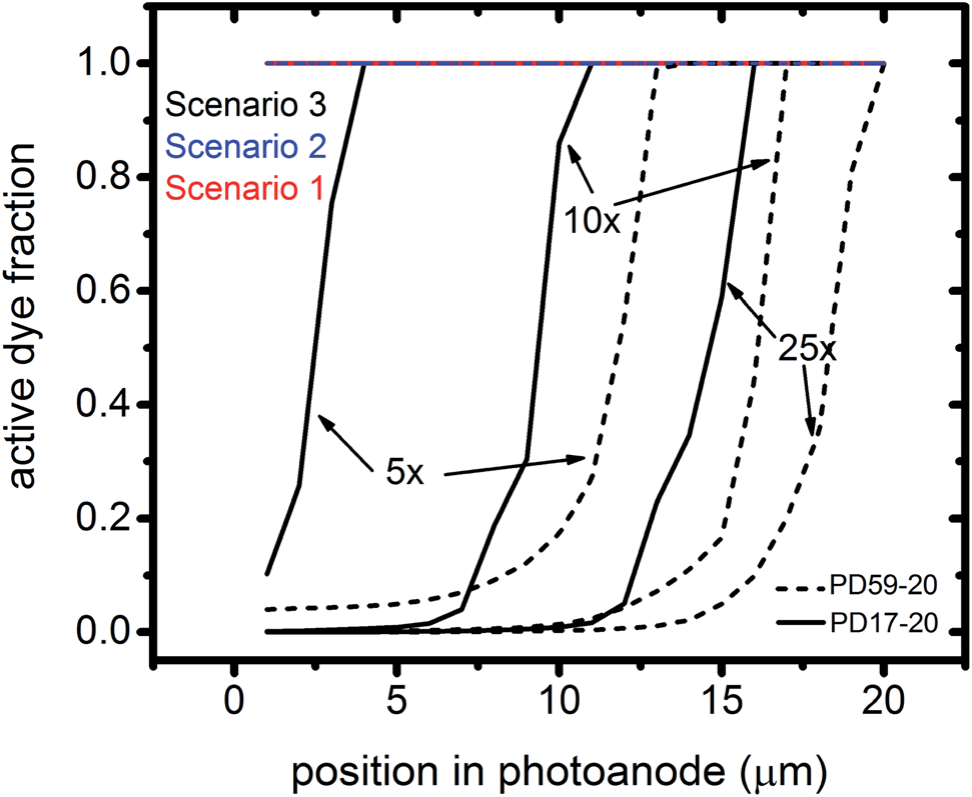
Profiles of the fraction of dyes that are photoactive within the pores for Sc1 and Sc2, which allow electrolyte–electron recombination, and Sc3, which does not, as a function of excitation frequency. The active dye fraction at 1× excitation frequency is 1.0 everywhere in the photoanode for both architectures and all 3 scenarios.

Photoexcited dyes are the source of electrons that undergo recombination with the electrolyte. Following charge injection, the dyes remain in their inactive, oxidized state (Dye^+^) until they react with 2I^−^ to form I_2_^−^ (Table 1, process 2). As shown in Fig. 8 and ESI, Fig. S2–S9,† under Sc3 there is a significant persistent population of Dye^+^ in the photoanode for excitation frequencies above 1 s^−1^. Because the oxidized dyes cannot undergo further excitation and charge injection, this has the effect of reducing the relative photosensitivity of the anode from 1 at 1× excitation frequency to 0.97, 0.69 and 0.37 at 5×, 10× and 25× excitation frequencies for PD17-20, and 0.74, 0.43 and 0.19 across the same series for PD59-20. On the other hand, under Sc1 and Sc2, the fraction of dyes that are active is high, and the relative photosensitivity stays at 1.

**Fig. 9:**
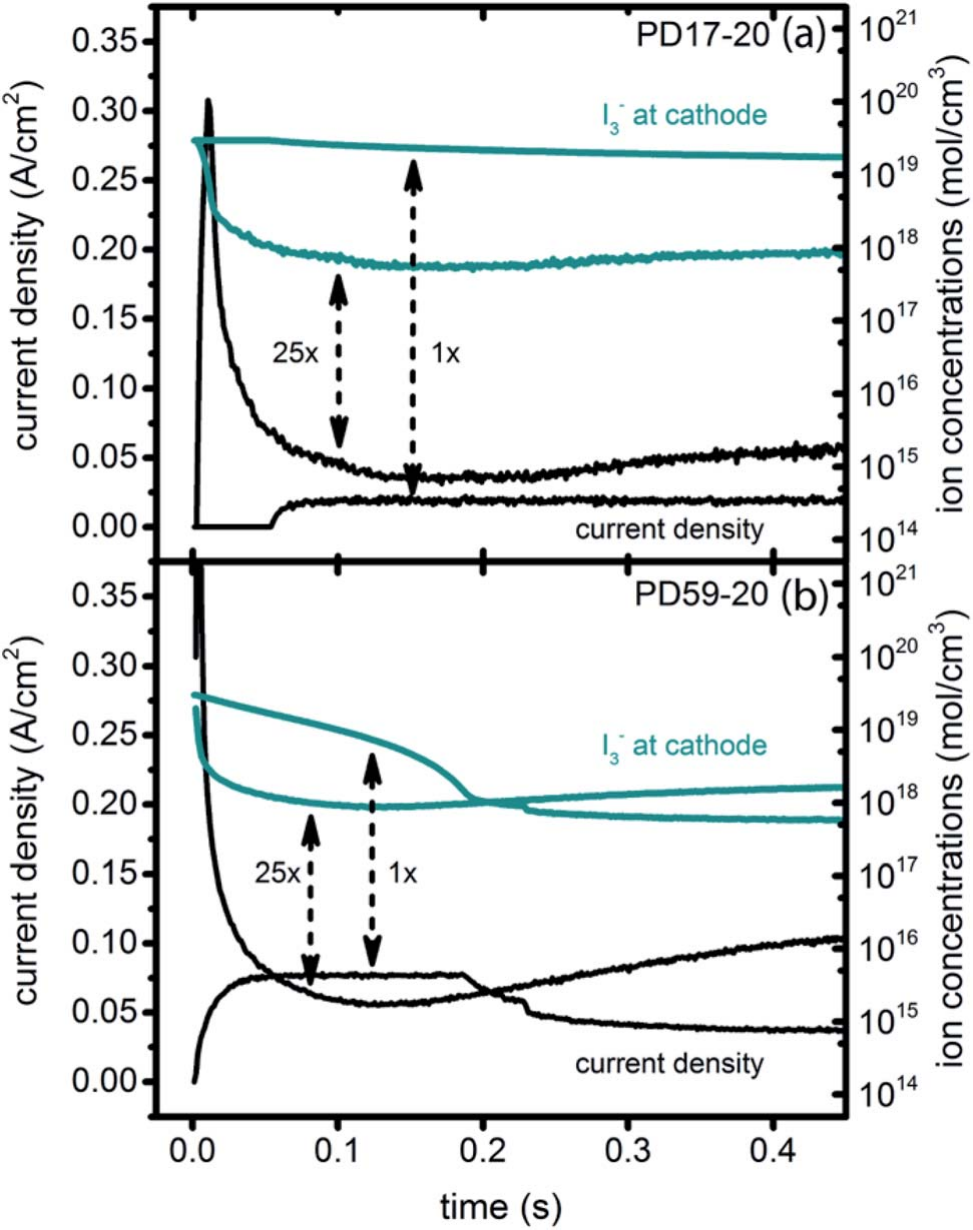
Current transients (left axis) and I_3_^−^ concentrations at the cathode (right axis) for Sc2. (a) PD17-20, 1× and 25× excitation frequencies; (b) PD59-20, 1× and 25× excitation frequencies.

Significant current transients are evident in Fig. 5 that cannot be explained by the electrolyte and interfacial simulation results described so far. The full composition *vs.* time maps for the systems presented in ESI, Fig. S2–S9† show that no transient concentration changes occur in the photoanode under any conditions. This is not the case for the bulk electrolyte near the cathode, however, where transients in the I_3_^−^ and I^−^ concentrations near the cathode are evident. Profiles for PD17-20 and PD59-20 using Sc2 are shown in Fig. 9. The current density transients for 25× excitation frequency at around 150 ms for both cases are accompanied by a depletion in local [I_3_^−^]. At 1× excitation frequency, the lower pore density case shows no transient and no I_3_^−^ depletion, and the high pore density case shows a more gradual decline in current density that tracks the decay in [I_3_^−^]. Further evidence that cathodic processes control current density is provided by the linear correlation between current density and [I_3_^−^] under all conditions except the PD17-20 architecture at 1× excitation frequency, as shown in ESI, Fig. S12.†

## Discussion

### Performance losses in DSCs

The primary goal of this work is to understand whether back electron transfer to electrolyte species is a source of performance losses in a simple photoelectrochemical energy conversion system, the DSC. The calculations show that electron losses due to recombination with I_2_, while large, do not significantly reduce photovoltage or photocurrent. The only factor found to do so that directly involves electrolyte in the photoanode is creation of a sink for I_3_^−^ on pore walls within photoanode, which reduces both the in-pore and cathodic sources of I^−^. The magnitudes of both relative photovoltages and photocurrents do have significant dependences on other characteristics of the system, however. They are dependent on pore geometry, with the 59% porosity case providing higher performance than the 17% case. They also depend on excitation frequency. The photocurrent ratio for the two geometries is 1 : 4 at 1× excitation frequency, as shown in the earlier study,^28^ and decreases to 1 : 2 above 5× excitation frequency. This trend occurs because the photocurrent is influenced in different ways by the photoanode (excitation) and cathode (consumption of electrons). The interplay between them is excitation frequency and transport-dependent. The cause of lower than theoretical performance, often attributed to electron losses alone, must have multiple physical origins.

The calculations provide information on how electrolyte–electron recombination influences DSC operation. It is a dominant process, but it does not affect the predicted current density (aside from the PD59-20, Sc2 case, where I_3_^−^ is depleted), and only modestly reduces the photovoltage. Current density is strongly influenced by cathodic reactions, however, which depend on the local concentration of I_3_^−^. Fig. 3 shows that this concentration is reduced near the cathode relative to its value in the bulk and in the pores for all 3 scenarios at all excitation frequencies. This is an incomplete picture, however, because the efficiency of the photoanode, where electrons are generated, depends on whether or not electrons are trapped by I_2_. As shown in Fig. 8, there is reduction in active dyes when no electrolyte–electron recombination occurs (Sc3), which acts to shut down the photoanode and suppress electron generation. It is possible that a significant population of oxidized dyes could make back electron transfer to Dye^+^, ordinarily very slow, kinetically important. If this occurs, for example by coulombic trapping or a hopping mechanism,^4,47^ it would be beneficial because it would increase dye cycling. However, the interfacial electron concentration, which ranges from 7 × 10^19^–8 × 10^20^ cm^−3^ for the cases studied here, is still less than the Dye^+^ concentration of about 10^21^ cm^−3^ and back electron transfer could not be a route to full activation of the photoanode. The entire photoanode remains active under Sc1 and Sc2, however a large fraction of the electrons are trapped by I_2_ and are swept into the electrolyte as I_2_^−^ rather than diffusing to the cathode. Despite these operational differences, the photocurrent is predicted to be nearly the same for all three scenarios. This indicates that for the conditions examined here, how much electrolyte–electron recombination occurs is not a dominant consideration for photocurrent generation. What is significant are the cathodic processes involving the electrolyte, which appear to be not just influential, but controlling (ESI, Fig. S12†).

That the details of the I^−^/I_3_^−^ electrolyte redox chemistry are central to DSC performance has been noted extensively in the literature. It has been proposed that the high efficiency of this redox couple in a device relative to other systems is due to the slow rate of electron scavenging by I_2_, governed by a small rate coefficient and a low I_2_ concentration,^24^ or by I_3_^−^.^48^ Another hypothesis is that Dye^+^ reduction by the electrolyte generates I_2_^−^, whose disproportionation products, I^−^ and I_3_^−^ either do not react with electrons released at the TiO_2_–dye–electrolyte interface (I^−^), or do so only very slowly.^39^ These proposals rest on the underlying assumption that preferential formation of I_3_^−^ is favored by the equilibrium reactions in process 1 under all operating conditions. In fact, the calculations show that this is not the case: the DSC is a driven system where equilibria are not necessarily maintained even when steady-state operating conditions are reached. The continuous cycling of dye excitations, charge injection and reduction of the oxidized dye depletes the electrolyte present in the pores of I^−^, and builds up I_3_^−^*via* I_2_^−^ disproportionation. The low concentration of I^−^ in the photoanode pores promotes dissociation of I_3_^−^ into I_2_ and I^−^, providing a dynamic and substantial source of I_2_ and consequently a high back electron transfer rate, as well as a significant local source of I^−^ that rapidly reduces oxidized dyes to make them available for photoexcitation and charge injection. The present work suggests that this chemistry may be more central to the usefulness of this redox couple.

### The electrolyte as an adaptive element that preserves system function

To gain deeper insights to the role of the electrolyte in the operation of this system, it is instructive to consider Fig. 3 and 6–8. Whether or not back electron transfer occurs, both previous work^28^ and the present study indicate that the photocurrent depends primarily on cathodic reactions, dye amounts in the pores, dye excitation frequency, and pore architecture. Electron losses are large, as shown in Fig. 7, but do not displace these controlling factors. Fig. 6 shows that the only signature of electron losses is a reduction of the photovoltage by a few mV. The model as constructed indicates that efficient recombination losses can be seen to be beneficial because they provide a means for all dyes to be active at all times within the photoanode. This compensation is due to changes in the rates of reactions in the electrolyte, which are situational, because of changes in concentrations as illustrated in Fig. 4. The dynamic changes in rates are fundamentally an adaptive response that confers functional resilience on the photoanode.

**Fig. 10:**
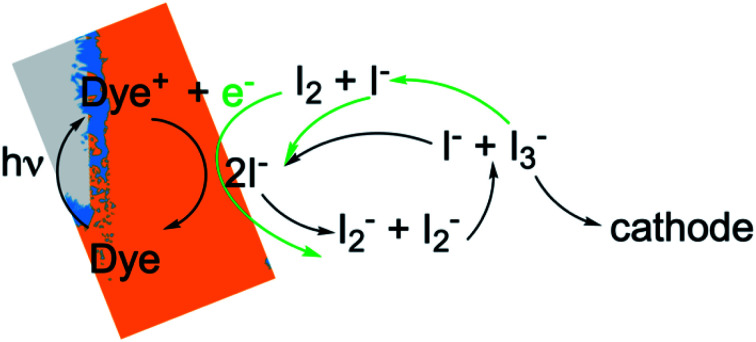
Schematic of the electrolyte reactions involved in reduction of Dye^+^. The paths indicated by black arrows correspond to Sc3. The green arrows show how the I_2_–electron recombination reactions from Sc1 and Sc2 are coupled to them.

The coupled chemical processes that enable this adaptivity are illustrated in Fig. 10. The pathways in black are those generally invoked in the literature, and the green pathways are those that are added in this work so that the electrolyte chemistry is described more completely. The key process is that depletion of I^−^ promotes formation of I^−^ within the pores through two reaction networks. One is the I_2_^−^ disproportionation step involving I_2_^−^ formed during Dye^+^ reduction, the other is I_3_^−^ dissociation in solution. The two networks are coupled by I_2_^−^ disproportionation which forms both I^−^ and I_3_^−^. This single point of coupling provides a feedback mechanism that is driven by dye excitation and oxidized dye reduction processes. The faster the dye excitation and charge injection, the faster I^−^ is consumed at the interface to generate I_2_^−^. The more I_2_^−^ there is, the faster the disproportionation reaction which has a second order dependence on its concentration. This does not fully replace I^−^, so leads to an acceleration of the rate of I_3_^−^ dissociation to form I_2_. I_2_ combines with electrons at the interface, forming I_2_^−^ and further increasing the disproportionation rate. The dual routes to I^−^ formation allow the system to cycle electrons locally *via* iodide ions, within the pores, at a rate sufficient to maintain photoanode efficiency without requiring transport of I_3_^−^ to the cathode. Thus, the electrolyte chemistry allows the photoanode's function to be resilient when driven by increasingly larger excitation frequencies and ever-increasing electrolyte–electron recombination. A process that should result in a net loss in the system's performance does not have this impact because it is not decoupled from a network of compensating chemical reactions.

That this electrolyte reaction mechanism is inherently adaptive is a prediction of this detailed computational study. Computational predictions need to be tested experimentally, and the simulation results point to useful measurements that will clarify their validity within a real system. As can be seen in Fig. 3, measurements of I^−^ and I_3_^−^ concentrations even within the pores will not enable the importance of the electron back reaction channel and hence the dual channel for I^−^ formation to be assessed. The presence of I_2_^−^, which is a sensitive probe of the I_2_ reaction channel, has not been detected.^24^ Consideration of the redox couple chemistry^12^ led to an estimate for the I_2_^−^ concentration of 3 μM at 10 mA cm^−2^ photocurrent. The simulation results show that it is much lower, in the range of 10^−8^ M within the pores at an excitation frequency of 1×, likely as a result of the high disproportionation rate within those small spaces. The simulations do predict a buildup of I_2_ within the pores at the highest excitation frequencies, however, which should be detectable if spectroscopies probing that region and not the bulk electrolyte are possible. Spectroscopic observations of the bulk electrolyte could be useful for a system under intense illumination. Any imbalances between I_3_^−^ and I^−^ due to the system being driven away from equilibrium as shown in Fig. 4 may be evident if the optical path length is sufficient. A potentially more definitive assessment would be *via* measurements of the presence or absence of oxidized dye in the photoanode *operando* as a function of excitation frequency, photoanode preparation techniques, and electrolyte composition. The simulations predict that Dye^+^ reduction is fast under all conditions if electrolyte–electron recombination is an important channel. If oxidized dyes are detected, they would indicate that the recombination and electrolyte chemical mechanisms described in the literature need to be refined. Finally, the detailed kinetics of the I_3_^−^ reduction reaction at the cathode are insufficiently understood. The assumptions in the present work lead to a conclusion that it is this reaction that ultimately limits photocurrent, however that could change if its mechanism proves to be significantly different than as described here.

## Conclusions

According to the literature, photocurrent and photovoltage in DSCs are limited by trapping of photogenerated electrons by species in the surrounding electrolyte. In this work, we have specifically examined whether this occurs by detailed multiscale reaction–diffusion simulations that connect nanoscale events to macroscale observables that allow this specific reaction channel to be turned on and off. The mechanism is constructed using data from the literature, and provide a means of predicting system characteristics based on what is known. We find that recombination events are dominant in the photoanode, however they do not significantly affect photovoltage or photocurrent *operando* because the electrolyte chemistry has an adaptive response that maximizes the efficiency of the photoanode under all conditions. The resilience of this photoanode–electrolyte combination to losses may explain the success of the I^−^/I_3_^−^ redox couple for this class of solar energy conversion systems. This adaptive response works by coupling two I^−^ regeneration pathways that are accelerated together as the dye cycling rate and the associated electron loss rate increase. While previously identified adaptive chemistries involve responses to changes in structure, the chemistry identified here enables the system's function to adapt to changing conditions. The detailed predictions from the calculations provide a basis for testing them *via* experimental studies, which would be invaluable for refinement of our understanding of how inefficiencies in this important class of solar-driven processes arise.

## Author contributions

F. A. H. conceived of this study, performed all calculations and data analysis, and prepared the manuscript and figures.

## Funding source

This material is based upon work supported by the U.S. Department of Energy, Office of Science, Office of Basic Energy Sciences, Chemical Sciences, Geosciences, and Biosciences Division, in the Solar Photochemistry Program under Contract No. DE-AC02-05CH11231.

## Conflicts of interest

There are no conflicts of interest to declare.

## Supplementary Material

SC-012-D1SC00384D-s001

SC-012-D1SC00384D-s002
