# Supplementary material for: Adaptive response by an electrolyte: resilience to electron losses in a dye-sensitized porous photoanode
Source: Chem Sci. 2021 Mar 25;12(17):6117–28. doi: 10.1039/d1sc00384d (PMC8098693; doi:10.1039/d1sc00384d)
Supplement: SC-012-D1SC00384D-s002 [file SC-012-D1SC00384D-s002.pdf]

Electronic Supplementary Material (ESI) for Chemical Science.
This journal is © The Royal Society of Chemistry 2021

Electronic Supplementary Information

Adaptive Response by an Electrolyte: Resilience to Electron Losses in a Dye-Sensitized Porous Photoanode

Frances A. Houle
Chemical Sciences Division, Lawrence Berkeley National Laboratory, 1 Cyclotron Road, Berkeley, CA 94720

Table of Contents

1. **Definitions of marker species and their uses**
2. **Sensitivity to magnitude of Keq**
3. **Reactant and product maps**
4. **Disproportionation events**
5. **Current vs time to 0.8s**
6. **Current density dependence at the cathode**
7. **References**

1. Definitions of marker species and their uses

The stochastic algorithm used for this work permits reaction steps in a mechanism to be written in a very flexible manner. It is especially useful to incorporate marker species, which are not chemically active, to track the occurrences of specific reactions as a function of location in the system. The markers used here are in italics as follows:

Electrolyte:
(Sc1 Sc2) $I_3^- \rightarrow I_2 + I^-$ + *I2formation*
(Sc1 Sc2) $I_2 + I^- \rightarrow I_3^-$
(all) 2 $I_2^- \rightarrow I^- + I_3^-$ + *disproportionation*

Photoanode interfacial layer
(all) Dye + hν → Dye^+ + electron + *excitation*
(all) $Dye^+ + I^-$ → Dye-I + *holeneutralization*
(all) Dye-I + I^- → Dye + I_2^-
(Sc1, Sc2) $I_2 + e^- \rightarrow I_2^-$ + *trap*
(Sc2) $I_2 + I^- \rightarrow I_3^-$ + *I3-formation*

Cathode:
$I_3^- + 2\ e^- \rightarrow 3\ I^-$ + *triiodidereduction*

The total numbers of markers as a function of position are analyzed to understand the relative importance of specific types of events as a function of Scenario, pore architecture and excitation frequency. The derivative of *triiodidereduction*, which tracks cathode chemistry, is used to calculate the current density.

2. Sensitivity to magnitude of Keq

The literature values for Keq for the reaction $I_3^- = I_2 + I^-$ span a range of 10^6 - 10^7 M^{-1} in acetonitrile.[1-5] In this work the lower value was used. To check the sensitivity of the simulation results to this assumption, simulations were performed for 2 randomly chosen cases, [PD17-20, Sc1, 5x] and [PD59-20, Sc2, 25x] using a value for Keq of $5.7\text{x}10^6$ M^{-1} as recommended in a recent review.[3] The results were broadly compared, and Figure S1 shows results for the marker species *trap*. The cumulative number of trapping events at 0.8s as a function of position in the photoanode pore is a sensitive test of whether the system's function is affected by the change in magnitude of Keq. The results show that the sensitivity in this range is minimal, with differences in event numbers at most at the parts per thousand level. Differences of this magnitude may also be a consequence of the random even selection sequence in the two cases.

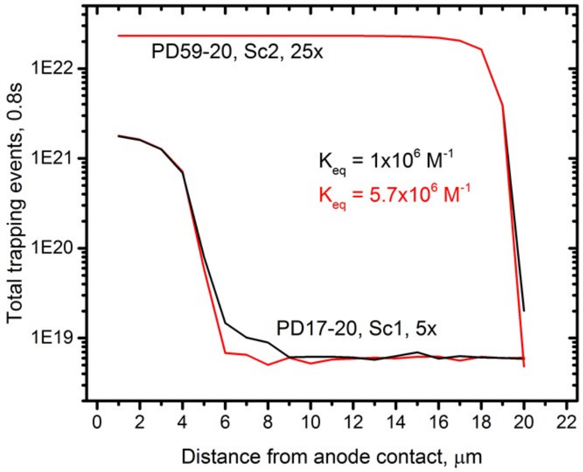

Figure S1. Comparison of data for the trap marker species as a function of simulation conditions for two values of Keq. A value of 10^6 M^{-1} was used in the main paper and all the other results in this ESI section.

3. Reactant and product maps

The simulations generate full time-dependent concentrations as a function of position in the system. The data are presented as maps in this section, comparing all three Scenarios for each combination of architecture and excitation frequency. The y axis for each panel is the position within the pore. The photoanode contact is at 0µm, the location of the interface between the photoanode and the bulk electrolyte is at 20µm, and the cathode is at 20 µm. Scenario 3 is where no electrolyte-electron recombination occurs, Scenario 1 allows recombination, and Scenario 2 allows recombination as well as $I_2 + I^-$ recombination at the interface.

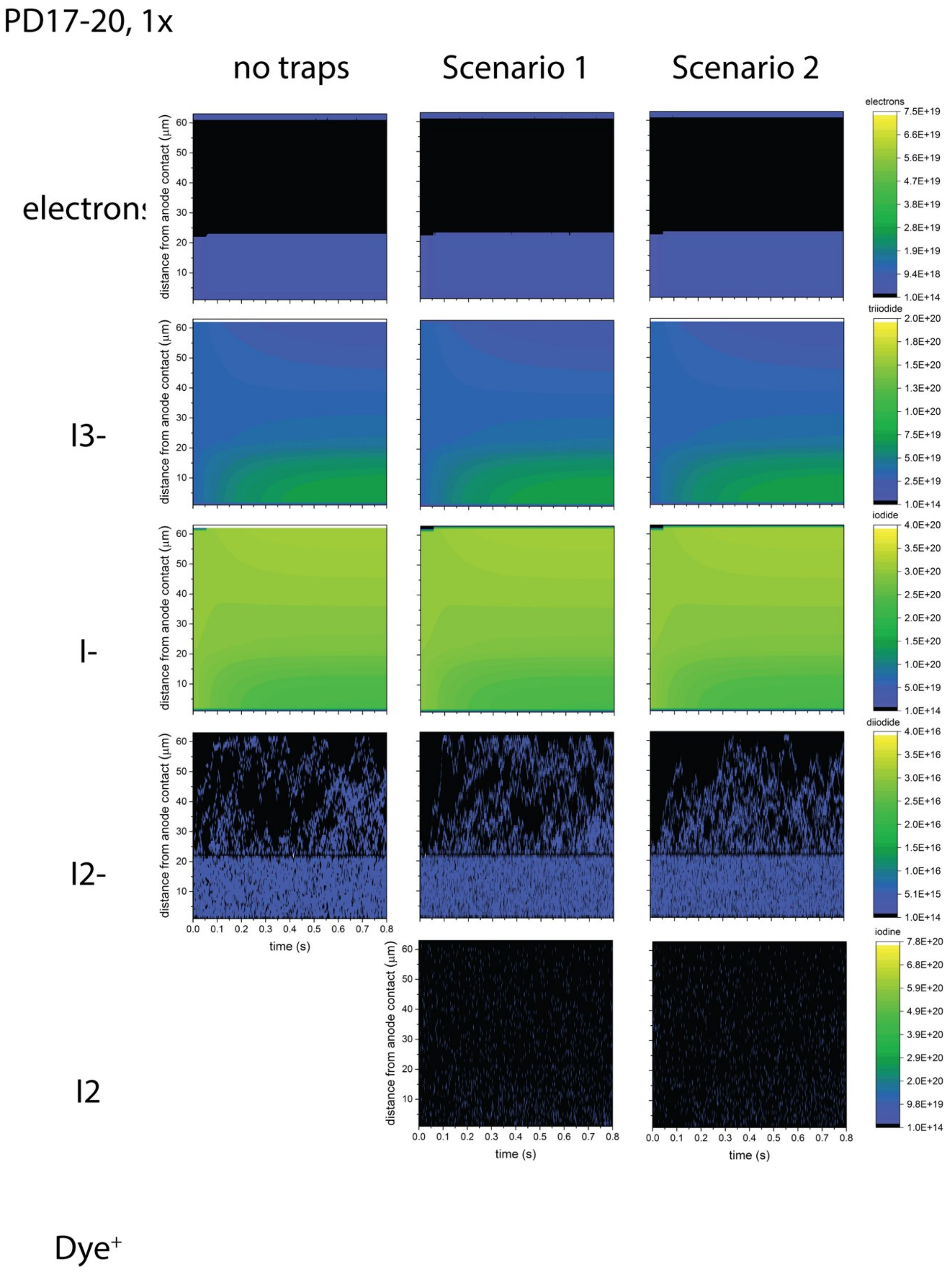
PD17-20, 1x
no traps
Scenario 1
Scenario 2
electrons
I3-
I-
I2-
I2
Dye+
distance from anode contact (μm)
time (s)
electrons
triiodide
iodide
diiodide
iodine

Figure S2

PD17-20, 5x

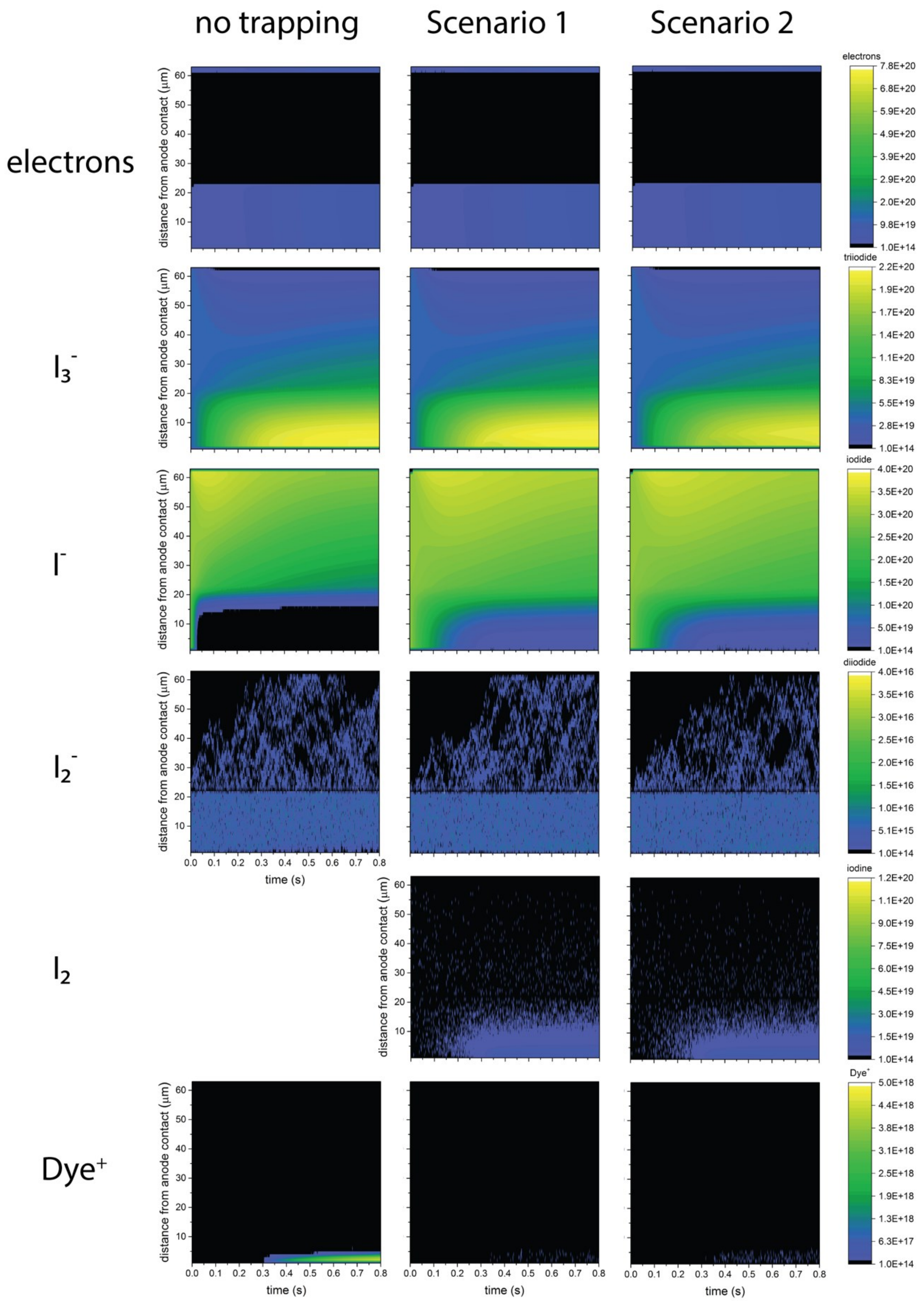

Figure S3

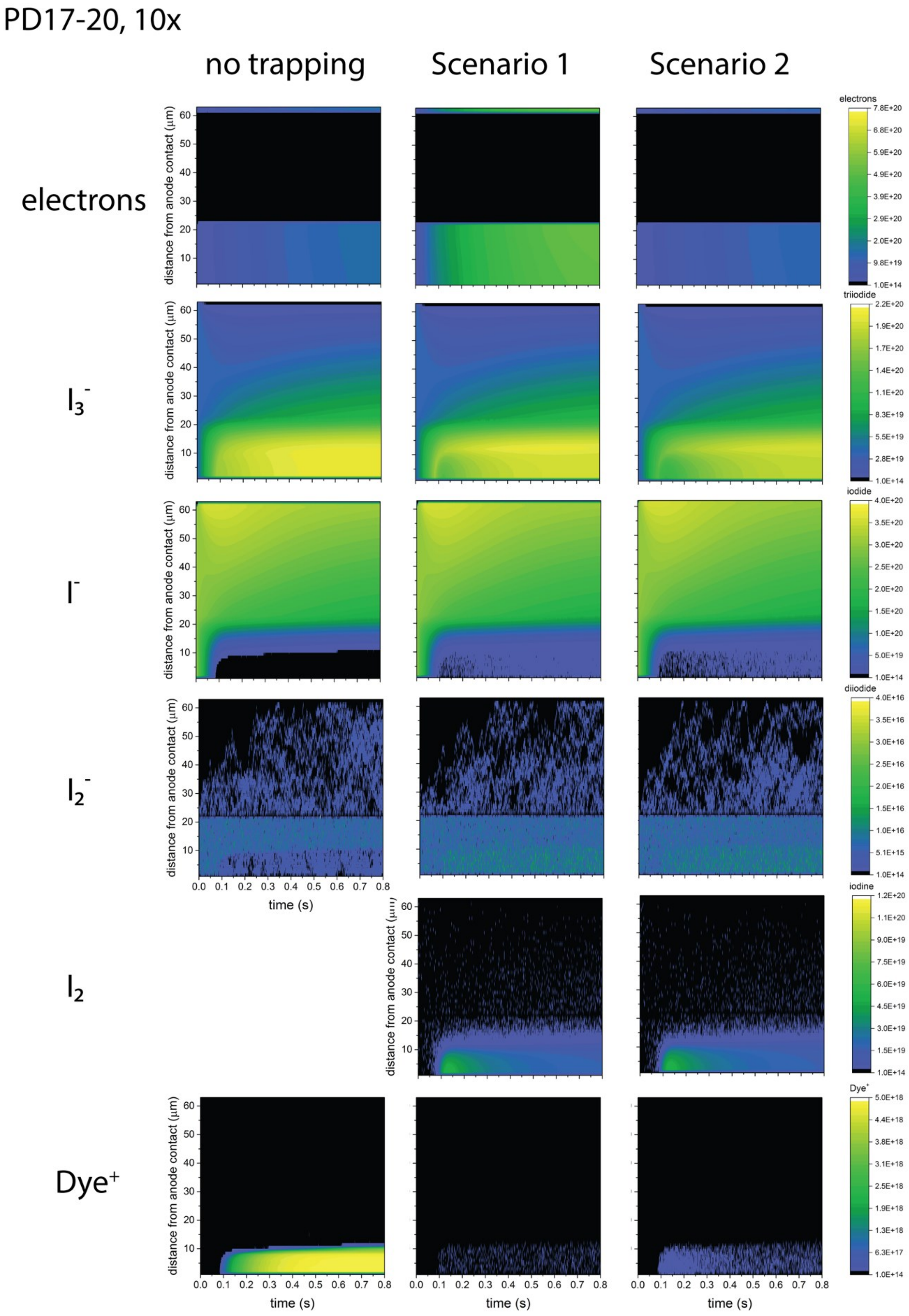

PD17-20, 10x
no trapping
Scenario 1
Scenario 2
electrons
I3-
I-
I2-
I2
Dye+
distance from anode contact (μm)
time (s)
electrons
triiodide
iodide
diiodide
iodine
Dye+

Figure S4

PD17-20, 25x

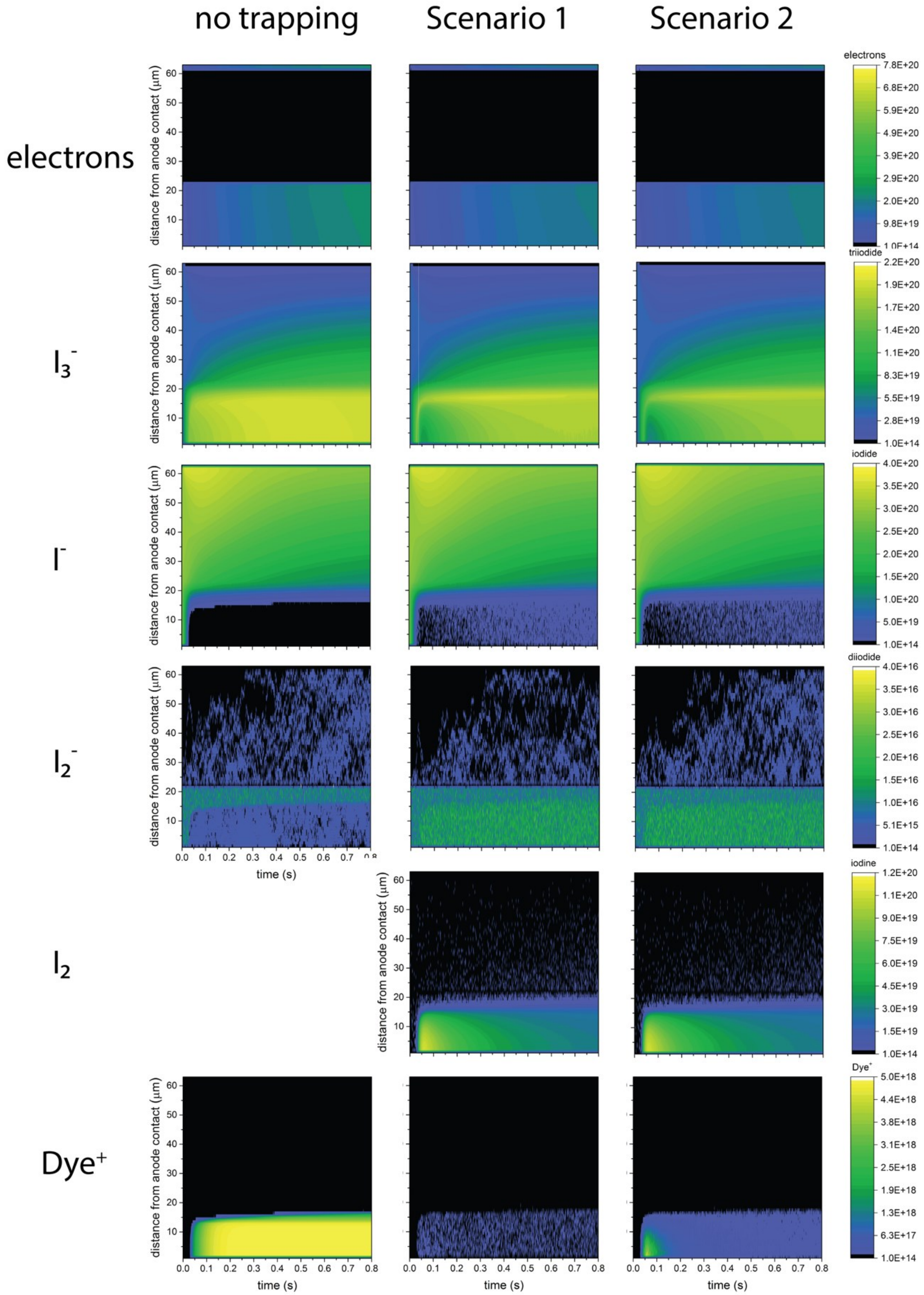

Figure S5

PD59-20, 1x

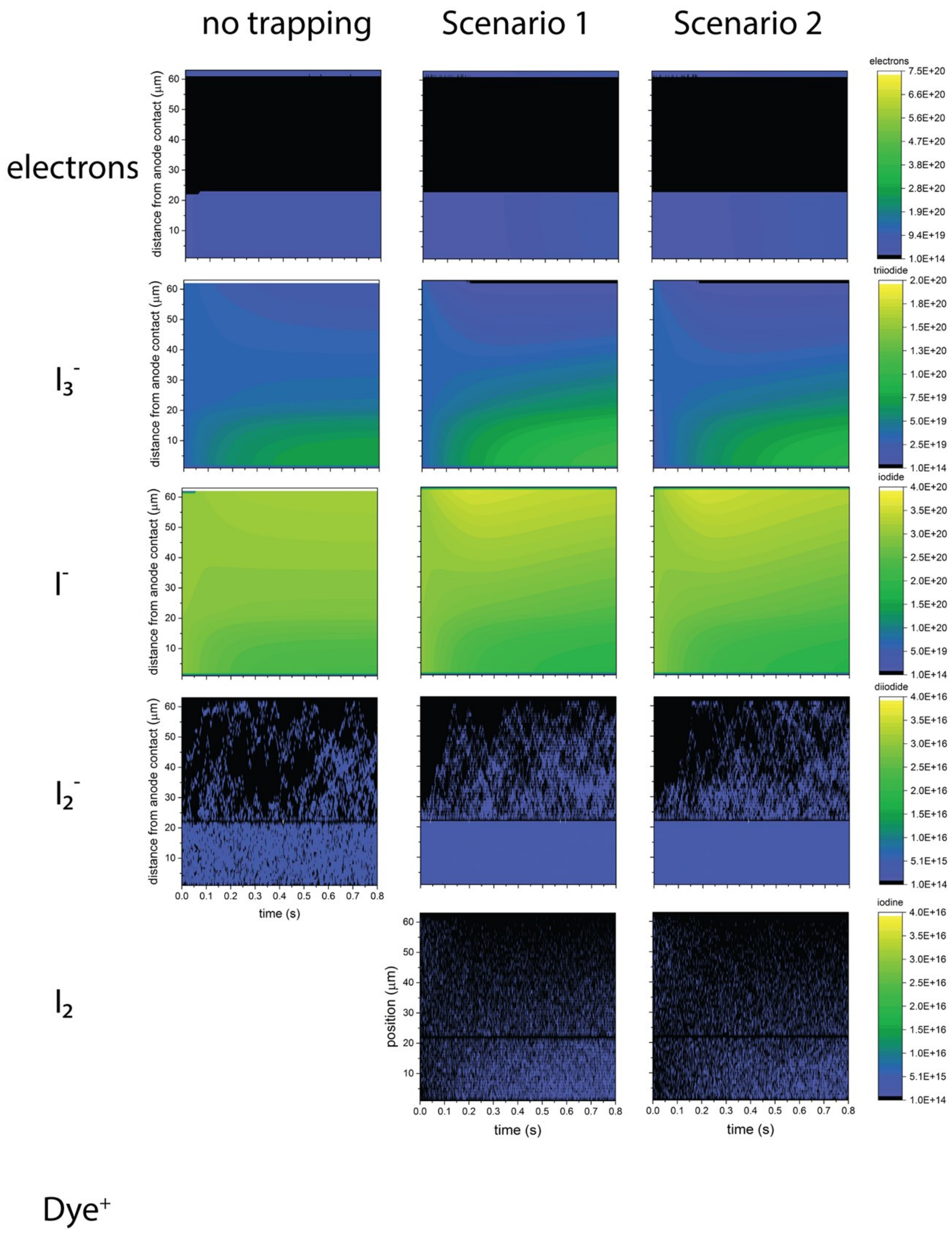

Figure S6

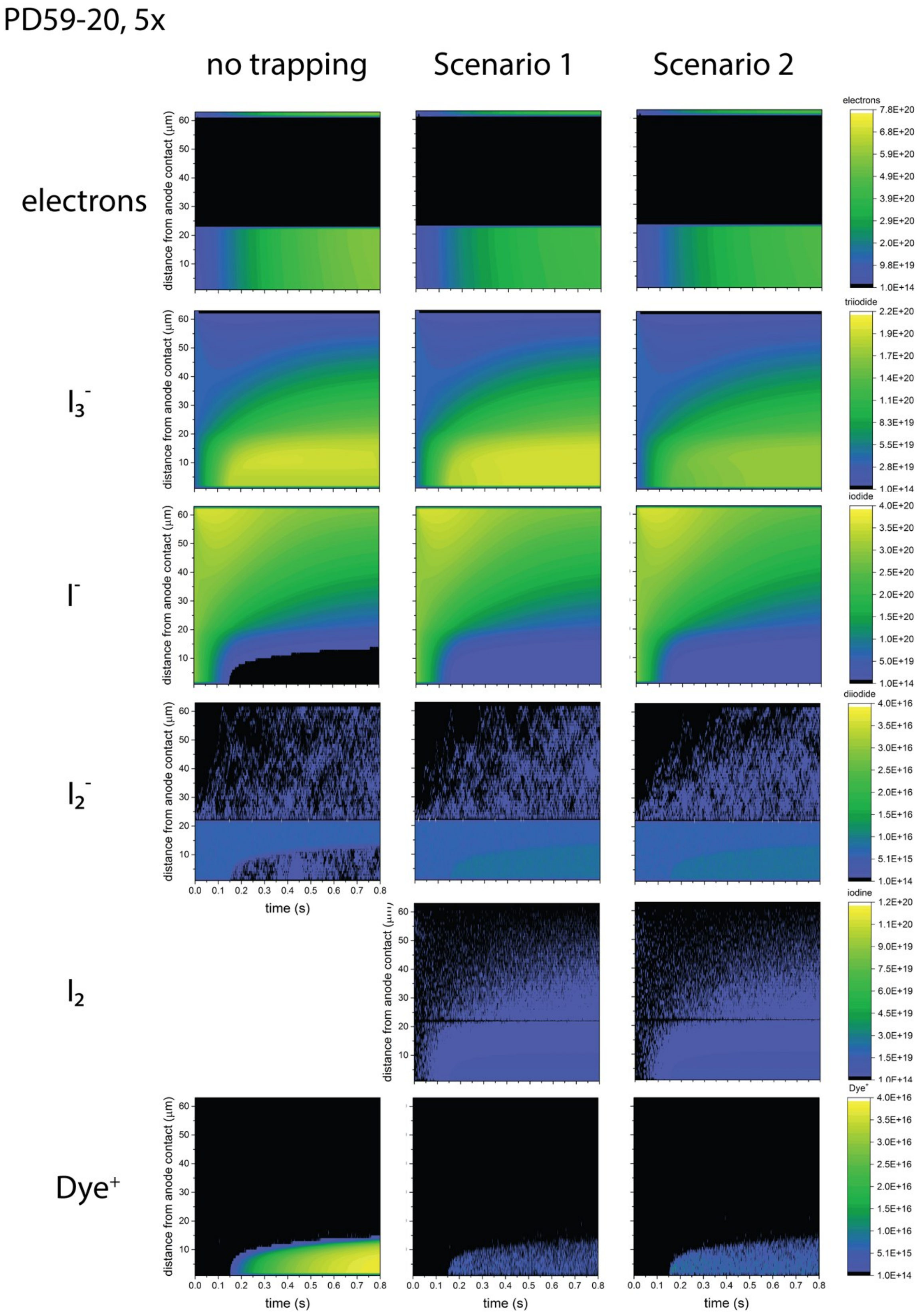

PD59-20, 5x
no trapping
Scenario 1
Scenario 2
electrons
I_3^-
I^-
I_2^-
I_2
Dye⁺
distance from anode contact (μm)
time (s)
electrons
7.8E+20
6.8E+20
5.9E+20
4.9E+20
3.9E+20
2.9E+20
2.0E+20
9.8E+19
1.0E+14
triiodide
2.2E+20
1.9E+20
1.7E+20
1.4E+20
1.1E+20
8.3E+19
5.5E+19
2.8E+19
1.0E+14
iodide
4.0E+20
3.5E+20
3.0E+20
2.5E+20
2.0E+20
1.5E+20
1.0E+20
5.0E+19
1.0E+14
diiodide
4.0E+16
3.5E+16
3.0E+16
2.5E+16
2.0E+16
1.5E+16
1.0E+16
5.1E+15
1.0E+14
iodine
1.2E+20
1.1E+20
9.0E+19
7.5E+19
6.0E+19
4.5E+19
3.0E+19
1.5E+19
1.0E+14
Dye⁺
4.0E+16
3.5E+16
3.0E+16
2.5E+16
2.0E+16
1.5E+16
1.0E+16
5.1E+15
1.0E+14

Figure S7

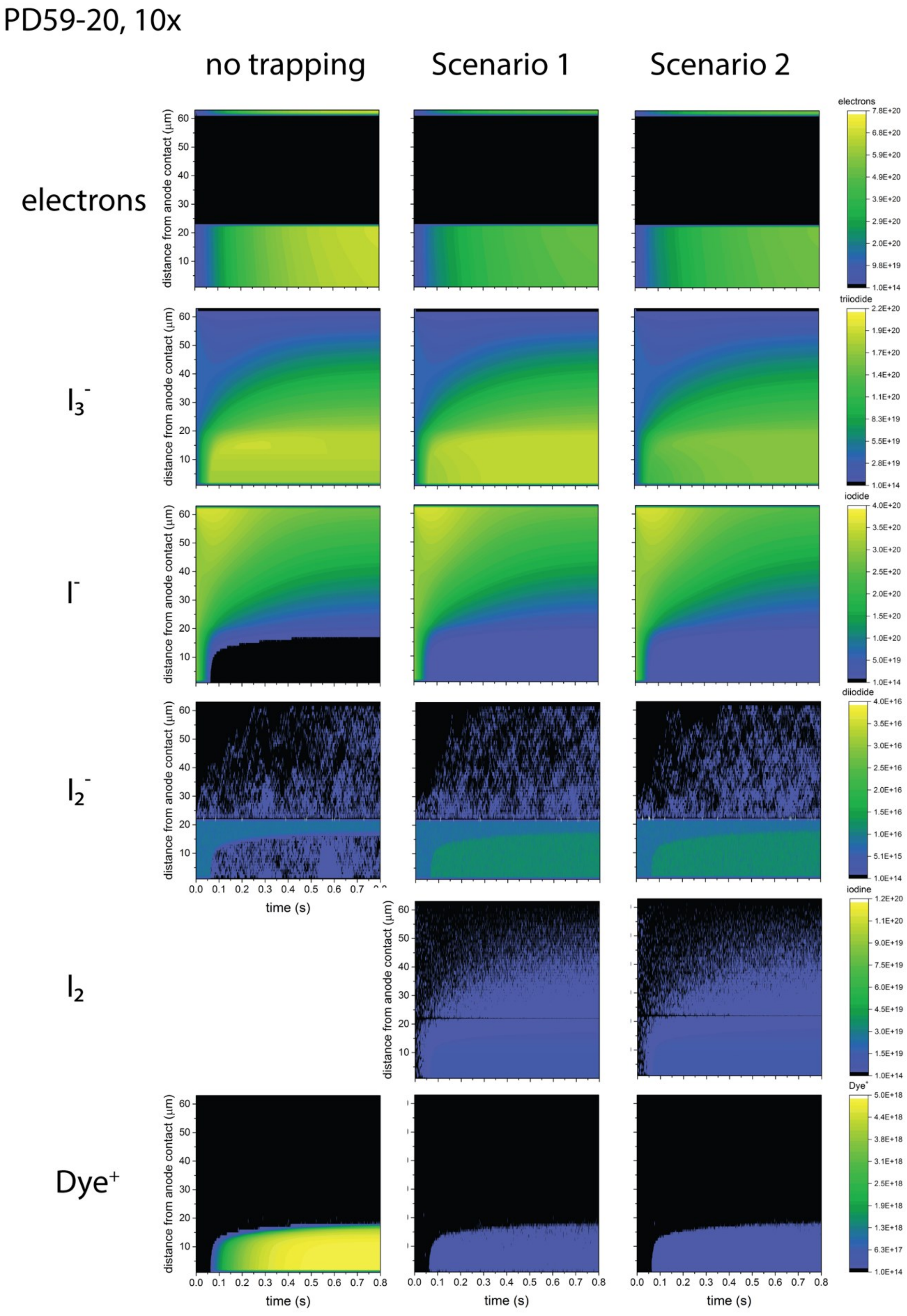

PD59-20, 10x
no trapping
Scenario 1
Scenario 2
electrons
I3-
I-
I2-
I2
Dye+
distance from anode contact (μm)
time (s)
electrons
triiodide
iodide
diiodide
iodine
Dye+

Figure S8

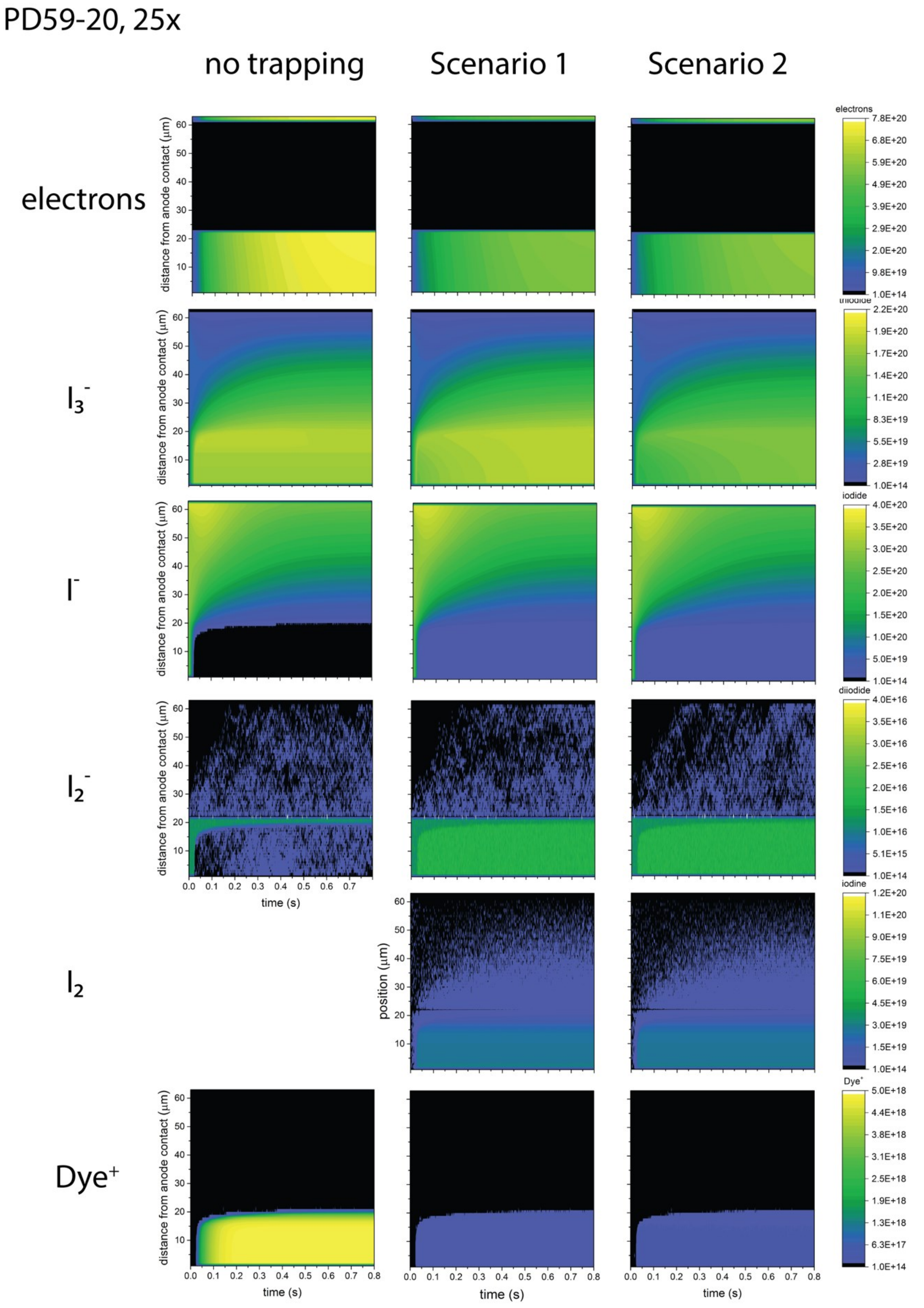
PD59-20, 25x
no trapping
Scenario 1
Scenario 2
electrons
I_3^-
I^-
I_2^-
I_2
Dye+
distance from anode contact (µm)
position (µm)
time (s)
electrons
triiodide
iodide
diiodide
iodine
Dye+

Figure S9

4. Disproportionation events

The marker species *disproportionation* tracks the total number and location of $I_2^- + I_2^-$ reactions as a function of time. I_2^- is formed by dye reduction and by recombination of electrons with I_2 (Scenarios 1 and 2). The disproportionation reaction generates I^- and I_3^- as products, and is central to I^- regeneration within the functioning photoanode pore.

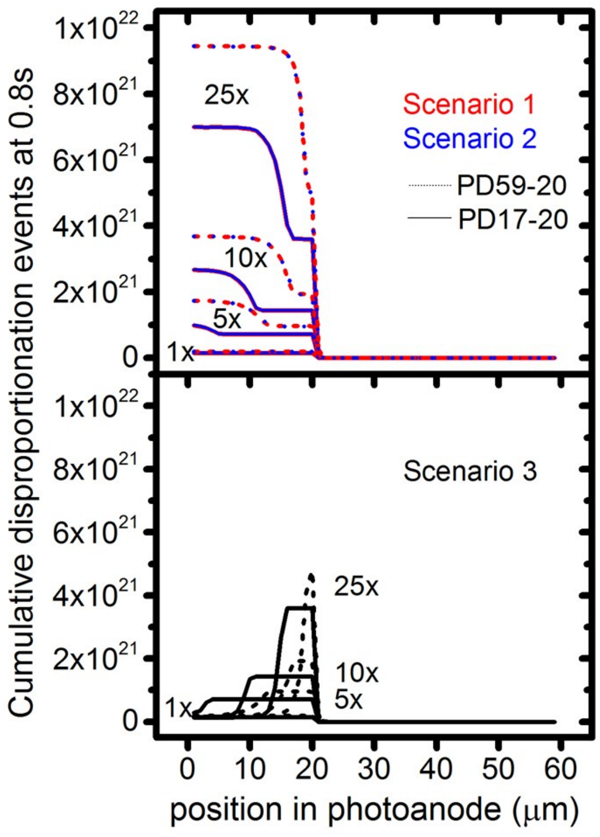

Figure S10. Cumulative disproportionation at 0.8s for the three Scenarios and two pore architectures.

5. Current vs time to 0.8s

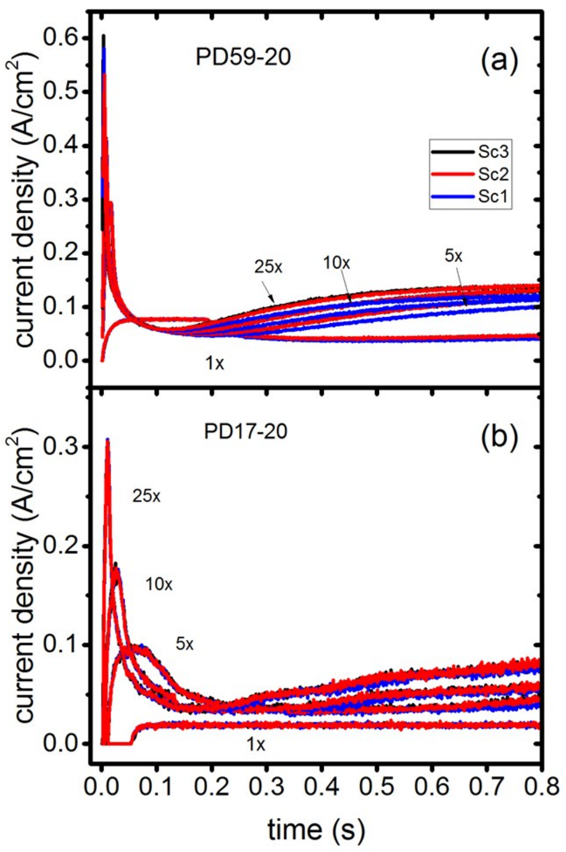

Figure S11. Photocurrent determined for all cases, where black is Scenario 3, blue is Scenario 2 and red is Scenario 1. (a) PD59-20; (b) PD17-20.

6. Current density at cathode

Although photoexcitation of the dyes generates electrons for the photocurrent, the current flow is controlled by the cathodic reaction $I_3^- + 2$ electrons $\rightarrow 3\ I^-$. The marker species *triiodidereduction* is used to calculate current density as a function of time. With the exception of the PD17-20 architecture at 1x excitation frequency, all cases have a linear correlation between I_3^- concentration near the cathode and current density, showing that it is the reduction reaction that limits the current density.

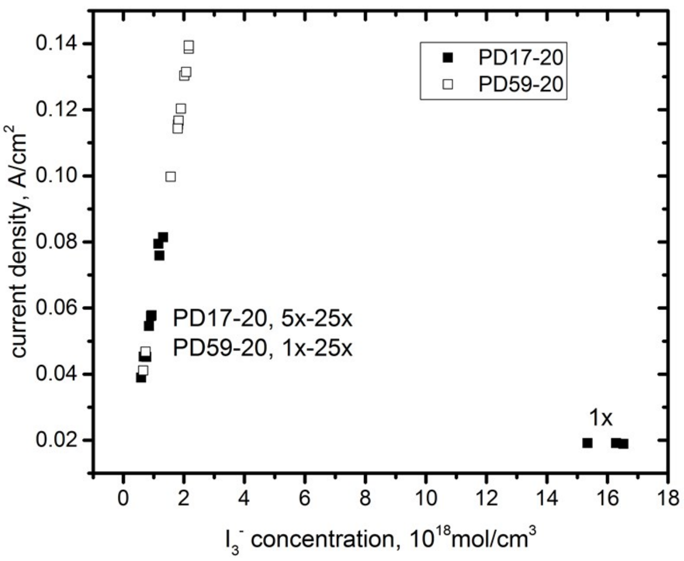

Figure S12. Correlation of current density with I_3^- concentration near the cathode for both architectures, all three Scenarios, and all excitation frequencies.

7. References

1. T. J. Barr and G. J. Meyer, *Acs Energy Lett*, 2017, **2**, 2335-2340.
2. F. G. K. Baucke, R. Bertram and K. Cruse, *J Electroanal Chem*, 1971, **32**, 247-&.
3. G. Boschloo and A. Hagfeldt, *Accounts Chem Res*, 2009, **42**, 1819-1826.
4. A. J. Parker, *J Chem Soc A*, 1966, DOI: DOI 10.1039/j19660000220, 220-&.
5. J. G. Rowley, B. H. Farnum, S. Ardo and G. J. Meyer, *J Phys Chem Lett*, 2010, **1**, 3132-3140.
